# Placental inflammatory cytokines mRNA expression and preschool children’s cognitive performance: a birth cohort study in China

**DOI:** 10.1186/s12916-023-03173-2

**Published:** 2023-11-20

**Authors:** Jixing Zhou, Juan Tong, Xue Ru, Yuzhu Teng, Menglong Geng, Shuangqin Yan, Fangbiao Tao, Kun Huang

**Affiliations:** 1 Department of Maternal, Child and Adolescent Health, School of Public Health, Anhui Medical University, Hefei, 230032 China; 2 Key Laboratory of Population Health Across Life Cycle (AHMU), MOE, Hefei, 230032 China; 3 NHC Key Laboratory of Study On Abnormal Gametes and Reproductive Tract, Hefei, 230032 China; 4 Anhui Provincial Key Laboratory of Population Health and Aristogenics, Hefei, 230032 China; 5 Maternal and Child Health Care Center of Ma’anshan, No 24 Jiashan Road, Ma’anshan 243011, Anhui, China; 6 Scientific Research Center in Preventive Medicine, School of Public Health, Anhui Medical University, Anhui Province, China

**Keywords:** Placenta, Cohort study, Cytokines, Cognition, Children

## Abstract

**Background:**

The immunologic milieu at the maternal–fetal interface has profound effects on propelling the development of the fetal brain. However, accessible epidemiological studies concerning the association between placental inflammatory cytokines and the intellectual development of offspring in humans are limited. Therefore, we explored the possible link between mRNA expression of inflammatory cytokines in placenta and preschoolers’ cognitive performance.

**Methods:**

Study subjects were obtained from the Ma’anshan birth cohort (MABC). Placental samples were collected after delivery, and real-time quantitative polymerase chain reaction (RT-qPCR) was utilized to measure the mRNA expression levels of IL-8, IL-1β, IL-6, TNF-α, CRP, IFN-γ, IL-10, and IL-4. Children’s intellectual development was assessed at preschool age by using the Wechsler Preschool and Primary Scale of Intelligence, Fourth Edition (WPPSI-IV). Multiple linear regression and restricted cubic spline models were used for statistical analysis.

**Results:**

A total of 1665 pairs of mother and child were included in the analysis. After adjusting for confounders and after correction for multiple comparisons, we observed that mRNA expression of IL-8 (*β* =  − 0.53; 95% CI, − 0.92 to − 0.15), IL-6 (*β* =  − 0.58; 95% CI, − 0.97 to − 0.19), TNF-α (*β* =  − 0.37; 95% CI, − 0.71 to − 0.02), and IFN-γ (*β* =  − 0.31; 95% CI, − 0.61 to − 0.03) in the placenta was negatively associated with preschoolers’ full scale intelligence quotient (FSIQ). Both higher IL-8 and IL-6 were associated with lower children’s low fluid reasoning index (FRI), and higher IFN-γ was associated with lower children’s working memory index (WMI). After further adjusting for confounders and children’s age at cognitive testing, the integrated index of six pro-inflammatory cytokines (index 2) was found to be significantly and negatively correlated with both the FSIQ and each sub-dimension (verbal comprehension index (VCI), visual spatial index (VSI), FRI, WMI, processing speed index (PSI)). Sex-stratified analyses showed that the association of IL-8, IFN-γ, and index 2 with children’s cognitive development was mainly concentrated in boys.

**Conclusions:**

Evidence of an association between low cognitive performance and high expression of placental inflammatory cytokines (IL-8, IL-6, TNF-α, and IFN-γ) was found, highlighting the potential importance of intrauterine placental immune status in dissecting offspring cognitive development.

**Supplementary Information:**

The online version contains supplementary material available at 10.1186/s12916-023-03173-2.

## Background

Healthy cognitive development in early childhood has a positive impact on an individual’s long-term life chances. Children with cognitive delays or cognitive impairments are more susceptible to enter negative developmental pathways, such as experiencing severe academic failure [1], depression and anxiety [2], low social development [3], and suicidal ideation [4].

The placenta, as the most important target organ connecting maternal–fetal communication, serves a key function in transmitting oxygen, exchanging nutrients and waste, and releasing hormones [5]. Optimal placental function is crucial for the trajectory of intrauterine fetal development, especially in the brain [6]. Placental inflammation is strongly implicated in fetal growth restriction, pre-eclampsia, miscarriage, and preterm delivery [7, 8]. Furthermore, placental inflammation may impact on fetal brain and neurological development, including early brain injury, pediatric stroke, and ventriculomegaly [9–12]. The pathway by which the placenta affects fetal brain development is known as the placenta–brain axis [13].

The elevated levels of circulating cytokines and their correlation with declined cognitive abilities in both individuals with schizophrenia and healthy controls suggest a possible contribution of inflammation to the exacerbation of neurocognitive dysfunction [14, 15]. Studies of the relationship between inflammatory biomarkers and cognitive developmental outcomes in children are receiving more attention [16–18]. Inflammation is a natural defense mechanism that allows body tissues to respond to potentially harmful stimuli [19]. During pregnancy, the placenta plays a crucial role in regulating the inflammatory process through immunomodulation and cell-to-cell communication between maternal and fetal tissues [20]. Animal models show that maternal immune activation-induced inflammatory processes have important negative effects on neuroplasticity and neurogenesis and are sufficient to impart lifelong psychiatric and neurologic disorders in offspring [21, 22]. Among the multiple inflammatory biomarkers of interest in the context of possible relationships with neurodevelopmental processes, the most frequently measured are cytokines [17]. Cytokines have a dual role the uterine immune response [23] and brain function [24, 25]. Cytokine imbalances can disrupt fetal development and chronically impair brain function, thereby may have a lasting effect on the neurological function of the offspring [26].

To our knowledge, there are studies that have focused on the links of maternal inflammatory cytokines during pregnancy with offspring’s neurodevelopmental outcomes, including cognitive performance [18, 27], neurodevelopmental delay [28], autism [29, 30], psychomotor development [31], and depression [32]. Although the findings of these studies are not consistent, it suggests that maternal biomarkers of inflammation such as IL-8, IL-6, IFN-γ, TNF-α, and CRP may be important biomarkers that affect children’s neurodevelopment [18, 27, 29, 31, 33]. However, only a few case series and case–control studies suggest that placental inflammation is related to early childhood neurological abnormalities, mainly manifesting as early brain damage [34]. Human data on the effects of inflammatory cytokines in utero on offspring’s brain and cognitive development remain scarce [34]. Moreover, the balance between pro- and anti-inflammatory cytokines has been identified as essential for individual’s normal cognitive function [35], and elevated production of inflammatory cytokines may be particular key mediators in modifying fetal brain development [36]. The integrated expression profile and interaction of pro/anti-inflammatory cytokines are critical for the normal function of the maternal and fetal immune systems [37].

Therefore, based on a Chinese birth cohort, we sought to test the association of placental inflammatory cytokines transcript expression and integrated index of pro- and anti-inflammatory cytokines with preschool children’s cognitive development and aimed to provide new clues to the impact of an adverse intrauterine immune environment on long-term cognitive development in children.

## Methods

### Study participants

This investigation was nested within the Ma’anshan Birth Cohort (MABC), which is a regional birth cohort study from Anhui, China. From May 2013 to September 2014, 3474 pregnant women who were first attending antenatal care at the Ma’anshan Maternal and Child Health Center in China were recruited.

The criteria for women’s inclusion included: ≥ 18 years of maternal age, within 14 weeks of gestation, living in Ma’anshan city for over 6 months, without mental illness, being able to understand and complete the questionnaires, and being willing to take part in follow-up assessments throughout the offspring’s childhood.

**Fig. 1:**
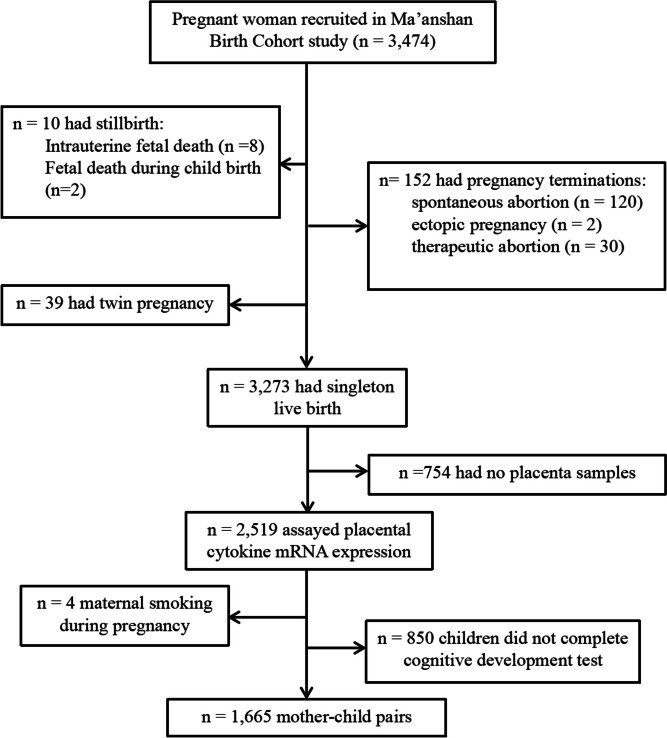
Flow chart of participants

We have set up the following exclusion criteria: (1) adverse pregnancy outcomes, including embryonic arrest, stillbirth, spontaneous abortion, therapeutic abortion, and ectopic pregnancy (*n* = 162); (2) multiple pregnancies (*n* = 39); (3) no placenta samples after delivery (*n* = 754); (4) missing data on preschoolers’ cognitive developmental assessments (*n* = 850); and (5) maternal smoking during pregnancy (*n* = 4). Finally, 1665 mother–child pairs were included in current analysis. A detailed flow chart of the inclusion of mother–child pairs is shown in Fig. 1.

### Collection and storage of placenta samples

Obstetricians/midwives collected placental tissues within half an hour after delivery (*n* = 2519). After washing the placenta with normal saline, an intact placental lobule free of maternal decidua, calcification, and fascia was extracted vertically from the full-thickness placentas in the position of 5-cm periumbilical and cut longitudinally into tissues smaller than ≤ 0.5 cm in size (each piece contained both maternal and fetal sides). The extracted sample was then immersed in RNAlater and saved at 4 °C overnight. The supernatant was drained the next morning, and the sample was transferred to a lyophilization tube and later stored at − 80 °C for testing.

### Assay of placental inflammatory cytokine mRNA expression

We used real-time quantitative polymerase chain reaction (RT-qPCR) to measure the expression of placental cytokine mRNA, including interleukin-8 (IL-8), IL-6, IL-1β, tumor necrosis factor-α (TNF-α), interferon-γ (IFN-γ), C-reactive protein (CRP), IL-4, and IL-10. The detailed detection approach was described in our prior study [38].

The TRIzol reagent (MRC Inc., Cincinnati, OH, USA) was used to isolate total RNA. A Nanodrop® ND-1000 (Nano Drop, USA) was used to evaluate all RNA quality, and the AMV Reverse Transcription System (Promega, USA) was used to reverse-transcribe total RNA (1.0 g) into cDNA with a value of 260/280 ≥ 1.8 in accordance with the manufacturer’s instructions. A 480 SYBR Green I kit (Roche Diagnostics GmbH, Mannheim, Germany) was used for real-time PCR. The amplification reactions were carried out on a Light Cycler® 480II instrument (Roche, Germany).

Ninety-five degrees Celsius for 10 min and 45 three-step cycles (95 °C for 15 s, 60 °C for 15 s, and 72 °C for 20 s) were procedures for the real-time PCR. Although we used 45 cycles, test wells with a CT > 40 were disregarded (per MIQE guidelines). Primer 5.0 was used to create an RT-qPCR primer. Sequences of the oligonucleotides utilized in qRT-PCR are displayed in Additional file 1: Table S1.

The 18S rRNA, used as an endogenous reference RNA, was used to normalize all RT-qPCR data. Delta cycle threshold (ΔCt) refers to the difference between the target mRNA and the normalized RNA (ΔCt = Ct mRNA-Ct normalized RNA). The detailed QC measures for this study are shown in Additional file 1: Table S2.

### Assessment of children’s cognitive development

The Wechsler Preschool and Primary School Scale of Intelligence, Fourth Edition of Chinese Version (WPPSI-IV CN) [39], which is extensively used in the field of cognitive function evaluation, was used to assess children’s cognitive development [40]. The following five subscales make up the WPPSI-IV: verbal comprehension index (VCI), visual spatial index (VSI), fluid reasoning index (FRI), working memory index (WMI), and processing speed index (PSI). The five domain subscales were used to determine the full-scale intelligence quotient (FSIQ).

Cognitive tests were administered to children aged 3.0–6.0 years by two professionally trained investigators in a quiet private room at the Ma’anshan Maternal and Child Health Center from June 2018 to January 2020. The age-standardized WPPSI-IV Chinese criteria were used to determine each child’s cognitive score. It is worth noting that two professionals taking the WPPSI were blinded to the placental cytokine data and important covariates.

### Covariates

We identified the confounders using literature review [38, 41, 42] and directed acyclic graph (DAG) [43] (Additional file 1: Fig. S1). The confounders in the current analysis included maternal age, pre-pregnancy BMI, maternal intelligence quotient (IQ), parity, family monthly income per capita, maternal metabolic dysfunctions during pregnancy, maternal fever during pregnancy, maternal infection or inflammation conditions during pregnancy, maternal alcohol use during pregnancy, father’s education level, placental efficiency, and children’s sex. Detailed information on confounders is presented in Additional file 1: Table S3. Early childhood information, including exclusive breastfeeding in the first 6 months, main caregivers before 3 years, time spent in outdoor activities, and screening was collected by questionnaires during the childhood follow-up survey. These variables were used in the sensitivity analyses.

### Statistical analysis

SPSS 23.0 software (*IBM*) and R software (version 4.2.1, R Core Team) were used for all statistical analyses, and *P*-values < 0.05 were defined as statistical significance.

To satisfy the requirements of normality and homogeneity of variance, the cytokine levels were translated to natural log-transform, which generated acceptable data skewness and kurtosis for further statistical analysis. For children’s and women’s Wechsler scale scores, the frequency distributions were moderately asymmetric, with skewness between − 1 and 1 for all variables and kurtosis between − 3 and 3 for all variables, indicating that the extreme values were not very different from those expected based on the normal data distribution. We used *t*-tests to compare the differences in cognitive developmental dimension scores between those with and without placental inflammatory cytokine data. Bivariate correlation using the Spearman correlation coefficient (*r*) was conducted to test the relationships between these placental cytokine expression measurements.

To fully explore the association between mRNA expression of inflammatory cytokines in the placenta and cognitive development in preschool children, we performed the following analyses.

First, the relationship between cytokines (IL-8, IL-1β, IL-6, TNF-α, CRP, IFN-γ, IL-10, and IL-4) and children’s cognitive score in each dimension (VCI, VSI, FRI, WMI, PSI, and FSIQ) was investigated separately using multiple linear regression models. The Benjamini–Hochberg procedure was used to adjust the false discovery rate (FDR) for multiple testing on eight inflammatory cytokines (IL-8, IL-1β, IL-6, TNF-α, CRP, IFN-γ, IL-10, and IL-4) [44].

Second, a significant interaction between inflammatory cytokines and sex was verified prior, and we further performed sex-stratified analyses in all adjusted models.

Third, we fitted two new indices, including an integrated index of anti-inflammatory cytokines (Index 1: IL-10 and IL-4) and an integrated index of pro-inflammatory cytokines (Index 2: IL-8, IL-6, TNF-α, IL-1β, CRP, and IFN-γ), to examine the association between integrated cytokines mRNA expression level and cognitive development. We summarized levels weighted by the number of cytokines in a given category based on the natural log-transformed values of each cytokine [the weights were 1/2 for cytokines *Index 1* (*n* = 2) and 1/6 for cytokines *Index 2* (*n* = 6). Then, multiple linear regression models were used to analyze the association between the two indices and children’s cognitive development scores.

Fourth, to examine the potential nonlinear relationship between placental inflammatory cytokines and children’s cognitive scores, a restricted cubic spline model was used to observe the association between each cytokine mRNA expression (IL-8, IL-1β, IL-6, TNF-α, CRP, IFN-γ, IL-10, and IL-4) and children’s cognitive development scores (VCI, VSI, FRI, WMI, PSI, and FSIQ).

Three sensitivity analyses were performed in all multiple linear regressions. First, considering that cognitive test age may affect children’s cognitive assessment performance, we further controlled for children’s age at cognitive testing to test the stability of the results. Second, breastfeeding duration [45], outdoor activities [46], screening [47], and main caregivers [48] may be important covariates that influence children’s cognitive development. Indeed, these factors will not confound the relation between exposure and outcome, but they are regarded as very important factors that related with children’s cognition function. Thus, we adjusted for these factors as precision variables to further explore the precision of the findings. Third, although existing studies of mRNA transcription support the use of 40 cycles or 45 cycles in human placental samples [49–51], we conducted further analyses of all data for Ct < 35 in each inflammatory cytokine, given the potential issues that might be posed by PCR Ct values all above 35 [IFN-γ was excluded because of its overall low expression in the placentas of this study, with high mean Ct (> 35)].

## Results

### Basic characteristics of included participants

In total, 1665 mother–child pairs were included in the analysis. Table 1 lists the basic characteristics of the included participants. The average maternal age was 26.4 years. Most of the pregnant women were primiparous (90.5%) and did not drink alcohol during pregnancy (92.4%), and the average maternal pre-pregnancy BMI was 20.9 kg/m^2^. The average gestational age was 39.1 weeks, and the average birth weight was 3383.5 g. The proportion of children born preterm was 3.2%. The mean age of the children’s cognitive assessment was 55.4 (± 6.8) months. Comparisons of the basic demographic characteristics of the included and excluded populations are shown in Additional file 1: Table S4.

**Table 1:** The basic characteristics of the included participants

Variables	Total (*n* = 1665)
Maternal characteristics	
Age at enrollment (years) (Mean ± SD)	26.4 ± 3.6
IQ (Mean ± SD)	96.3 ± 11.0
Parity [*n* (%)]	
Nulliparous	1507 (90.5)
Multipara	158 (9.5)
Pre-pregnancy BMI (kg/m^2^) (mean ± SD)	20.9 ± 2.8
Maternal metabolic dysfunctions during pregnancy [*n* (%)]	
Yes	285 (17.1)
No	1380 (82.9)
Maternal infection or inflammation conditions during pregnancy [*n* (%)]	
Yes	145 (8.7)
No	1520 (91.3)
Maternal fever during pregnancy [*n* (%)]	
Yes	213(12.8)
No	1452(87.2)
Alcohol use during pregnancy [*n* (%)]	
No	1539 (92.4)
Yes	126 (7.6)
Father’s characteristics	
Father’s educational levels [*n* (%)]	
Junior high school or below	223 (13.4)
Senior middle school	487 (29.2)
Junior college or above	955 (57.4)
Family monthly income per capita, RMB/yuan [*n* (%)]	
< 2500	440 (26.4)
≥ 2500	1225 (73.6)
Placental characteristics	
Placental weight (g) (mean ± SD)	587.9(167.3)
Placental efficiency (mean ± SD)	6.12(1.9)
Children’s characteristics	
Sex [*n* (%)]	
Boys	859 (51.6)
Girls	806 (48.4)
Birth weight (g) (mean ± SD)	3383.5 ± 427.3
Gestational weeks (mean ± SD)	39.1 ± 1.3
Preterm birth [*n* (%)]	54 (3.2)
Children’s age at cognition testing (months) (mean ± SD)	55.4 (6.8)
Exclusive breastfeeding for the first six months [*n* (%)]	
Yes	174 (10.5)
No	1491 (89.5)
Main caregivers before 3 years [*n* (%)]	
Parents	857 (51.5)
Grandparents	808 (48.5)
Average screen time per day [*n* (%)]	
≤ 1 h/day	568 (34.1)
> 1 h/day	1097 (65.9)
Average outdoor activity time per day [*n* (%)]	
≤ 1 h/day	402 (24.1)
> 1 h/day	1263 (75.9)

*IQ* Intelligence quotient, *SD* Standard deviation

### Distribution of placental cytokine expression and children’s cognitive performance

The Ct for 18S, IL-8, IL-1β, IL-6, TNF-α, CRP, IFN-γ, IL-4, and IL-10 are shown in Additional file 1: Table S5. All cytokines had a strong positive correlation between each other. The Spearman correlation coefficients for each inflammatory cytokine are shown in Additional file 1: Fig. S2.

The mean age of children was 55.4 months. The average scores (SD) of children in this study on VCI, VSI, FRI, WMI, PSI, and FSIQ were 109.0(12.7), 106.6(12.8), 104.8(11.4), 103.4(11.9), 101.9(10.9), and 107.8(11.2), respectively (Additional file 1: Table S6). There were no significant differences in FSIQ between children without placental data and children with placental data. The sub-dimensions of children’s cognitive scores in both groups are shown in Additional file 1: Table S6.

### Associations between placental inflammatory cytokines expression and children’s cognitive performance

**Fig. 2:**
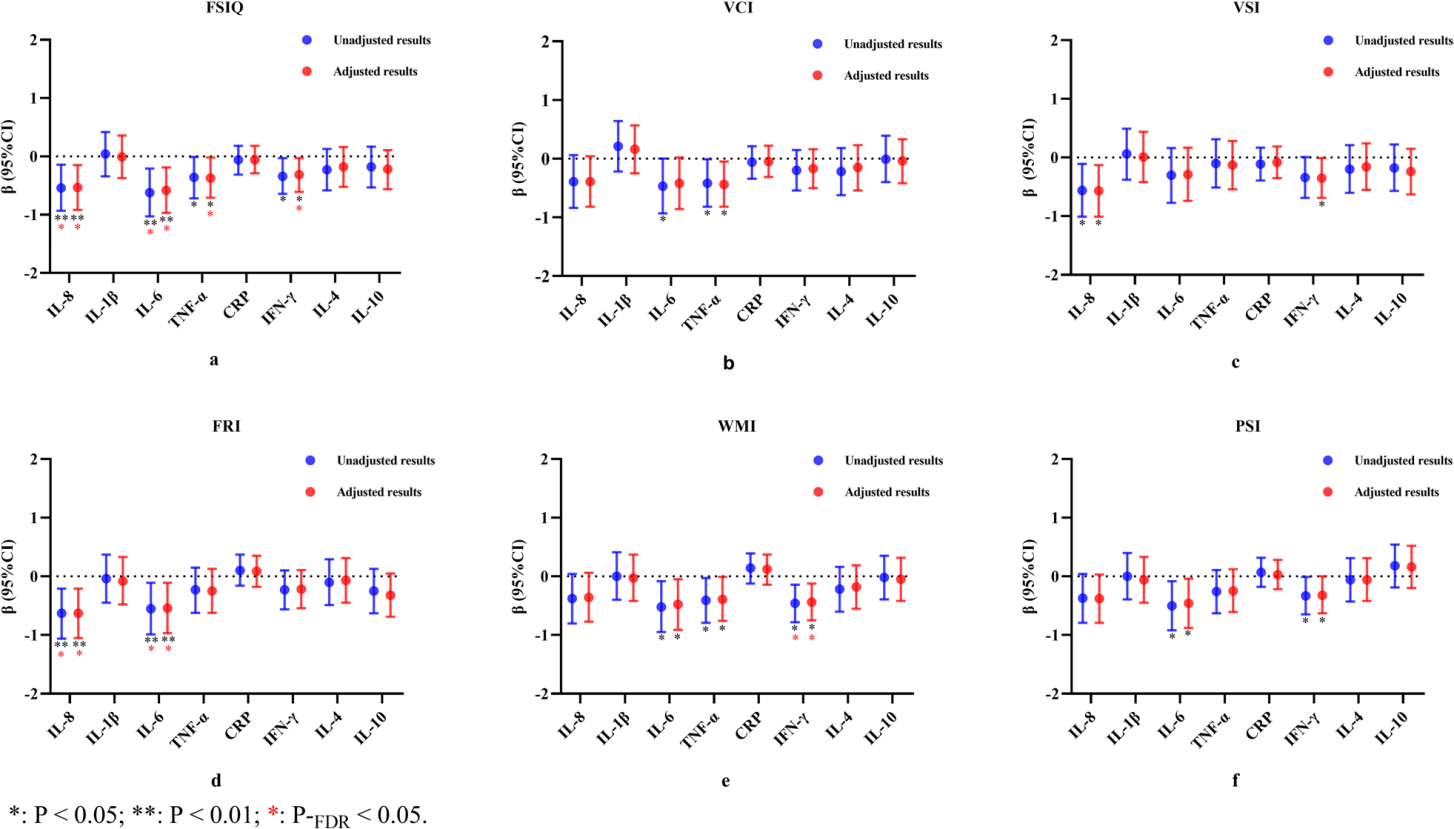
The associations between placental inflammatory cytokines mRNA expression (IL-8, IL-1β, IL-6, TNF-α, CRP, IFN-γ, IL-10 and IL-4) and children’s cognitive performance (VCI, VSI, FRI, WMI, PSI and FSIQ) by linear regression model

The associations between placental cytokine mRNA expression and cognitive test scores among preschool children are shown in Fig. 2. After adjusting for confounders, we observed significant associations between multiple placental cytokines mRNA expression and children’s cognitive scores (Table 2). For the pro-inflammatory chemokine IL-8, it was found to be negatively associated with preschoolers’ WPPSI scores on VSI (*β* =  − 0.57; 95% CI, − 1.01 to − 0.13), FRI (*β* =  − 0.63; 95% CI, − 1.05 to − 0.21), FSIQ (*β* =  − 0.53; 95% CI, − 0.92 to − 0.15). In pro-inflammatory cytokines, for IL-6, it was negatively associated with scores on FRI (*β* =  − 0.54; 95% CI, − 0.97 to − 0.11), WMI (*β* =  − 0.48; 95% CI, − 0.91 to − 0.05), PSI (*β* =  − 0.46; 95% CI, − 0.88 to − 0.04), and FSIQ (*β* =  − 0.58; 95% CI, − 0.97 to − 0.19). For TNF-α, it was negatively associated with scores on VCI (*β* =  − 0.44; 95% CI, − 0.82 to − 0.05), WMI (*β* =  − 0.39; 95% CI, − 0.76 to − 0.01), and FSIQ (*β* =  − 0.37; 95% CI, − 0.71 to − 0.02). For IFN-γ, it was negatively associated with scores on VSI (*β* =  − 0.35; 95% CI, − 0.69 to − 0.01), WMI (*β* =  − 0.44; 95% CI, − 0.75 to − 0.12), PSI (*β* =  − 0.32; 95% CI, − 0.63 to 0.00), and FSIQ (*β* =  − 0.31; 95% CI, − 0.61 to − 0.03). We did not observe any significant linear association between anti-inflammatory cytokines (IL-4 and IL-10) and preschoolers’ cognitive performance.

**Table 2:** Multiple linear regression analysis of the relationship between placental cytokine mRNA expression and children’s cognitive performance

Cytokines	Participants	VCI	VSI	FRI	WMI	PSI	FSIQ
		Adjusted *β* (95%CI)					
IL-8							
	Total^a^	-0.39(-0.82,0.04)	-0.57(-1.01,-0.13)*	-0.63(-1.05,-0.21)**^★^	-0.36(-0.77,0.06)	-0.38(-0.79,0.03)	-0.53(-0.92,-0.15)**^★^
	Boys^b^	-0.29(-0.95,0.36)	-0.97(-1.64,-0.31)**^★^	-0.65(-1.27,-0.03)*	-0.75(-1.37,-0.14)*^★^	-0.64(-1.23,-0.05)*	-0.77(-1.35,-0.18)*
	Girls^b^	-0.49(-1.05,0.07)	-0.26(-0.85,0.33)	-0.67(-1.24,-0.10)*	-0.05(-0.61,0.51)	-0.16(-0.73,0.41)	-0.37(-0.86,0.12)
IL-1β							
	Total^a^	0.16(-0.25,0.57)	0.01(-0.42,0.44)	-0.08(-0.48,0.33)	-0.03(-0.42,0.37)	-0.06(-0.45,0.33)	-0.01(-0.37,0.36)
	Boysb	0.10(-0.52,0.71)	0.14(-0.48,0.76)	-0.17(-0.74,0.40)	-0.25(-0.82,0.33)	0.01(-0.53,0.56)	-0.05(-0.60,0.50)
	Girls^b^	0.22(-0.34,0.77)	-0.15(-0.74,0.43)	-0.01(-0.58,0.56)	0.14(-0.41,0.70)	-0.14(-0.71,0.42)	0.00(-0.487,0.486)
IL-6							
	Total^a^	-0.42(-0.86,0.02)	-0.29(-0.74,0.17)	-0.54(-0.97,-0.11)*^★^	-0.48(-0.91,-0.05)*	-0.46(-0.88,-0.04)*	-0.58(-0.97,-0.19)**^★^
	Boys^b^	0.02(-0.65,0.69)	-0.68(-1.36,0.00)*	-0.81(-1.44,-0.18)*	-0.83(-1.45,-0.20)**^★^	-0.45(-1.06,0.15)	-0.61(-1.20,-0.01)*
	Girls^b^	-0.86(-1.44,-0.28)**^★^	0.03(-0.58,0.65)	-0.30(-0.89,0.29)	-0.13(-0.71,0.46)	-0.46(-1.05,0.12)	-0.58(-1.09,-0.07)*
TNF-α							
	Total^a^	-0.44(-0.82,-0.05)*	-0.13(-0.54,0.28)	-0.25(-0.62,0.13)	-0.39(-0.76,-0.01)*	-0.25(-0.61,0.12)	-0.37(-0.71,-0.02)*^★^
	Boys^b^	-0.04(-0.63,0.55)	-0.08(-0.68,0.52)	0.09(-0.46,0.63)	-0.56(-1.11,-0.01)*	-0.18(-0.71,0.34)	-0.20(-0.72,0.33)
	Girls^b^	-0.84(-1.34,-0.33)**^★^	-0.23(-0.76,0.31)	-0.56(-1.08,-0.05)*	-0.20(-0.71,0.32)	-0.31(-0.82,0.21)	-0.55(-1.00,-0.10)*
CRP							
	Total^a^	-0.05(-0.31,0.22)	-0.08(-0.35,0.19)	0.09(-0.18,0.35)	0.12(-0.14,0.37)	0.03(-0.22,0.28)	-0.06(-0.29,0.18)
	Boys^b^	-0.24(-0.63,0.15)	-0.07(-0.46,0.33)	-0.03(-0.40,0.34)	-0.06(-0.42,0.31)	-0.31(-0.66,0.04)	-0.24(-0.59,0.10)
	Girls^b^	0.15(-0.20,0.51)	-0.06(-0.44,0.31)	0.26(-0.11,0.63)	0.32(-0.04,0.68)	0.46(0.10,0.83)*	0.16(-0.16,0.47)
IFN-γ							
	Total^a^	-0.17(-0.50,0.16)	-0.35(-0.69,-0.01)*	-0.22(-0.54,0.11)	-0.44(-0.75,-0.12)**^★^	-0.32(-0.63,0.00)*	-0.31(-0.61,-0.03)*^★^
	Boys^b^	-0.04(-0.53,0.45)	-0.25(-0.74,0.25)	-0.09(-0.55,0.38)	-0.57(-1.03,-0.11)*^★^	-0.46(-0.90,-0.01)*	-0.37(-0.80,0.07)
	Girls^b^	-0.30(-0.74,0.14)	-0.46(-0.93,0.01)	-0.35(-0.80,0.10)	-0.30(-0.75,0.14)	-0.16(-0.61,0.29)	-0.28(-0.67,0.11)
IL-4							
	Total^a^	-0.15(-0.54,0.23)	-0.16(-0.55,0.24)	-0.07(-0.45,0.31)	-0.18(-0.55,0.19)	-0.06(-0.42,0.31)	-0.18(-0.52,0.16)
	Boys^b^	-0.03(-0.61,0.55)	-0.15(-0.73,0.44)	0.19(-0.35,0.74)	-0.31(-0.85,0.24)	-0.01(-0.53,0.51)	-0.22(-0.74,0.30)
	Girls^b^	-0.27(-0.77,0.23)	-0.16(-0.69,0.37)	-0.30(-0.83,0.23)	-0.04(-0.55,0.47)	-0.07(-0.59,0.46)	-0.12(-0.57,0.32)
IL-10							
	Total^a^	-0.04(-0.42,0.33)	-0.24(-0.63,0.15)	-0.32(-0.69,0.05)	-0.05(-0.42,0.32)	0.16(-0.20,0.52)	-0.22(-0.56,0.11)
	Boys^b^	-0.05(-0.61,0.51)	-0.25(-0.82,0.31)	-0.56(-1.09,-0.04)*	-0.32(-0.84,0.21)	-0.51(-0.55,0.45)	-0.39(-0.89,0.14)
	Girls^b^	-0.06(-0.56,0.44)	-0.23(-0.76,0.30)	-0.02(-0.55,0.51)	0.24(-0.27,0.75)	0.38(-0.14,0.91)	-0.06(-0.50,0.39)

*CI* Confidence interval, *IL* Interleukin, *CRP* C-reactive protein, *TNF-α* Tumor necrosis factor-alpha, *IFN-γ* Interferon-gamma, *FSIQ* Full-scale intelligence quotient, *VCI* Verbal comprehension index, *VSI* Visual spatial index, *FRI* Fluid reasoning index, *WMI* Working memory index, *PSI* Processing speed index

^a^Adjusted for maternal age, maternal IQ, family monthly income per capita, pre-pregnancy BMI, parity, maternal metabolic dysfunctions, maternal fever during pregnancy, maternal infection or inflammation conditions during pregnancy, maternal alcohol use during pregnancy, father’s education level, placental efficiency, and children’s sex

^b^Adjusted for maternal age, maternal IQ, family monthly income per capita, pre-pregnancy BMI, parity, maternal metabolic dysfunctions, maternal fever during pregnancy, maternal infection or inflammation conditions during pregnancy, maternal alcohol use during pregnancy, father’s education level, and placental efficiency

^*^p < 0.05; **p < 0.01;^**★**^*P*-_FDR_ < 0.05

When *p*-values were adjusted for multiple testing corrections, we still observed significant negative correlations of IL-8 (*P*-_*FDR*_ = 0.02), IL-6 (*P*-_*FDR*_ = 0.02), TNF-α (*P*-_*FDR*_ = 0.04), and IFN-γ (*P*-_*FDR*_ = 0.05) all with FSIQ. Furthermore, IL-8 was negatively associated with FRI (*P*-_*FDR*_ = 0.02), IL-6 was negatively associated with FRI (*P*-_*FDR*_ < 0.05), and IFN-γ was negatively associated with WMI (*P*-_*FDR*_ < 0.05) (Fig. 2 and Table 2).

In addition, we added a model that forced all cytokines to be included in one model and found that IL-8, IL-6, and IFN-γ were all significantly associated with certain dimensions of cognitive development scores (Additional file 1: Table S7).

### Sex-stratified associations between placental inflammatory cytokines expression and children’s cognitive performance

The association between placental inflammatory cytokines mRNA expression*sex and children’s cognitive performance is presented in Additional file 1: Table S8. The results of the analysis stratified by sex are shown in Table 2. After adjusting for confounders and performing multiple testing corrections, we observed that IL-6 was negatively associated with WMI in boys (*β* =  − 0.83; 95% CI, − 1.45 to − 0.20; *P*-_FDR_ < 0.05) and VCI in girls (*β* =  − 0.86; 95% CI, − 1.44 to − 0.28; *P*-_FDR_ < 0.05). IL-8 was negatively associated with VSI (*β* =  − 0.97; 95% CI, − 1.64 to − 0.31; *P*-_FDR_ < 0.05) and WMI (*β* =  − 0.75; 95% CI, − 1.37 to − 0.14; *P*-_FDR_ < 0.05) in boys. TNF-α was negatively associated with VCI in girls (*β* =  − 0.84; 95% CI, − 1.34 to − 0.33; *P*-_FDR_ < 0.01). IFN-γ was negatively associated with WMI in boys (*β* =  − 0.57; 95% CI, − 1.03 to − 0.11; *P*-_FDR_ < 0.05).

### Associations between summary index of cytokines and children’s cognitive performance

Linear regression analyses of the association between the summary index of cytokines and preschoolers’ cognitive scores are shown in Table 3. It was observed that index 2 (integrated index of six pro-inflammatory cytokines) was negatively correlated with FSIQ (*β* =  − 0.65; 95% CI, − 1.16 to − 0.15; *P* = 0.01).

**Table 3:** Associations between placental summary index of cytokines and children’s cognitive performance

Summary index of cytokines	Participants	VCI	VSI	FRI	WMI	PSI	FSIQ
		Adjusted *β* (95%CI)					
Index 1							
	Total^a^	-0.17(-0.67,0.33)	-0.34(-0.85,0.18)	-0.34(-0.84,0.16)	-0.20(-0.68,0.29)	0.09(-0.39,0.57)	-0.34(-0.78,0.10)
	Boys^b^	-0.07(-0.83,0.68)	-0.35(-1.12,0.41)	-0.36(-1.07,0.36)	-0.55(-1.25,0.16)	-0.05(-0.74,0.63)	-0.53(-1.21,0.14)
	Girls^b^	-0.28(-0.93,0.38)	-0.33(-1.02,0.36)	-0.27(-0.96,0.41)	0.16(-0.49,0.82)	0.26(-0.42,0.94)	-0.15(-0.73,0.43)
Index 2							
	Total^a^	-0.46(-1.03,0.11)	-0.52(-1.11,0.07)	-0.49(-1.05,0.07)	-0.51(-1.06,0.04)	-0.48(-1.02,0.06)	-0.65(-1.16,-0.15)^*^
	Boys^b^	-0.27(-1.13,0.58)	-0.62(-1.49,0.24)	-0.50(-1.31,0.31)	-1.05(-1.85,-0.25)^**^	-0.86(-1.63,-0.08)^*^	-0.85(-1.61,-0.09)^*^
	Girls^b^	-0.66(-1.42,0.09)	-0.47(-1.27,0.33)	-0.47(-1.24,0.30)	0.02(-0.74,0.78)	-0.08(-0.84,0.68)	-0.49(-1.16,0.17)

*CI* Confidence interval, *IL* Interleukin, *CRP* C-reactive protein, *TNF-α* Tumor necrosis factor-alpha, *IFN-γ* Interferon-gamma, *FSIQ* Full-scale intelligence quotient, *VCI* Verbal comprehension index, *VSI* Visual spatial index, *FRI* Fluid reasoning index, *WMI* Working memory index, *PSI* Processing speed index, *Index 1* Integrated index of anti-inflammatory cytokines (IL-10 and IL-4), *Index 2* Integrated index of pro-inflammatory cytokines (IL-8, IL-6, TNF-α, IL-1β, CRP, and IFN-γ)

^a^Adjusted for maternal age, maternal IQ, family monthly income per capita, pre-pregnancy BMI, parity, maternal metabolic dysfunctions, maternal fever during pregnancy, maternal infection or inflammation conditions during pregnancy, maternal alcohol use during pregnancy, father’s education level, placental efficiency, and children’s sex

^b^Adjusted for maternal age, maternal IQ, family monthly income per capita, pre-pregnancy BMI, parity, maternal metabolic dysfunctions, maternal fever during pregnancy, maternal infection or inflammation conditions during pregnancy, maternal alcohol use during pregnancy, father’s education level, and placental efficiency

^*^p < 0.05; ^**^p < 0.01

Stratified analysis by sex showed that index 2 was mainly negatively associated with boys’ cognitive development (FSIQ, WMI, and PSI) (Table 3).

### Test for non-linear relationship between placental cytokines mRNA expression and preschool children’s total cognitive performance

**Fig. 3:**
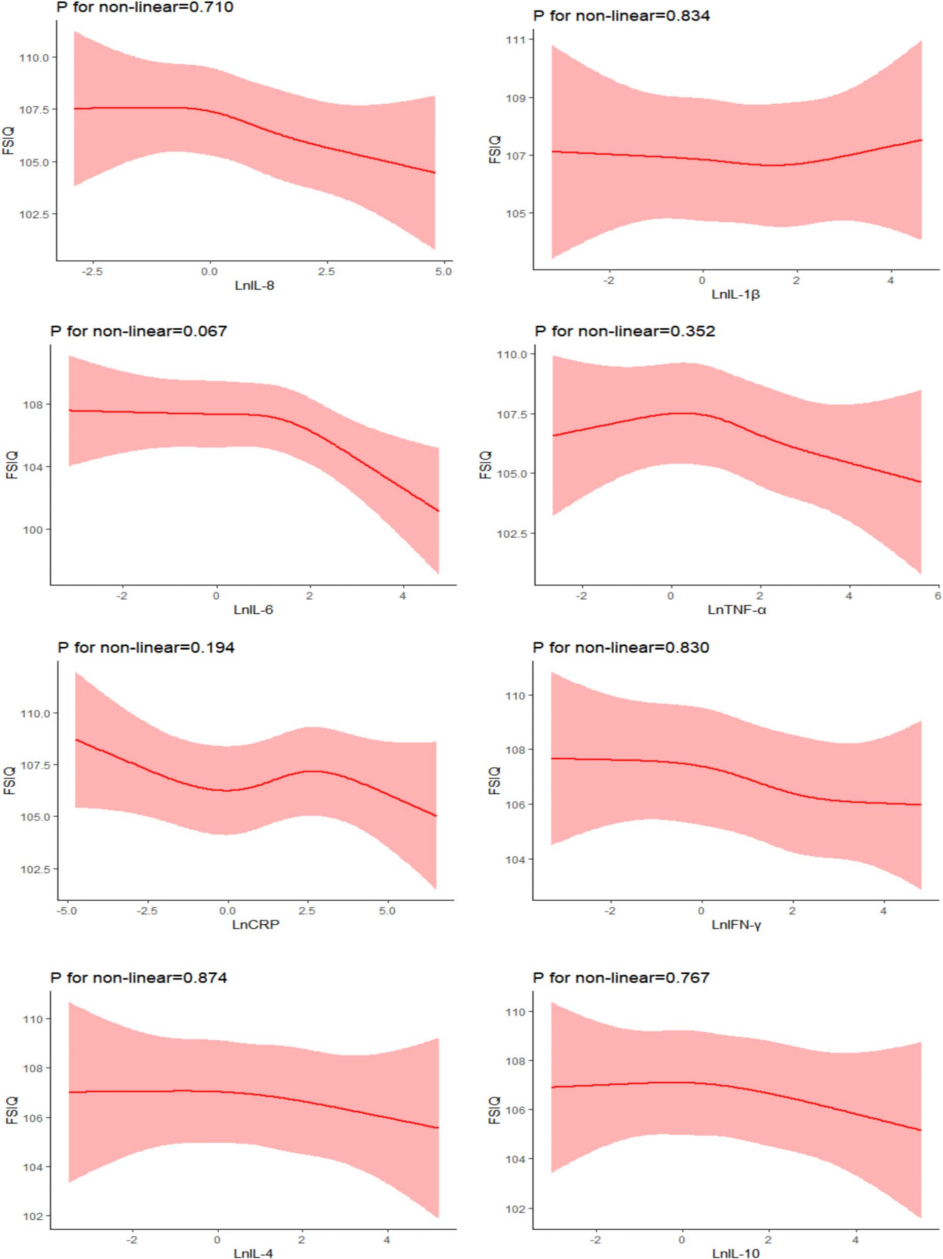
Restricted cubic spline analysis of the association between placental inflammatory cytokine mRNA expression (IL-8, IL-1β, IL-6, TNF-α, CRP, IFN-γ, IL-10 and IL-4) and children’s FSIQ

After adjusting for the confounders, we did not find any significant non-linear association of placental inflammatory cytokines mRNA expression (IL-8, IL-1β, IL-6, TNF-α, IFN-γ, IL-10, and IL-4) with FSIQ (Fig. 3), suggesting that these four cytokines (IL-1β, CRP, IL-10, and IL-4) did not have any significant associations (linear and nonlinear) with FSIQ in preschool children. In further exploring the nonlinear associations of placental inflammatory cytokine mRNA expression with cognitive subdimensions (VCI, VSI, FRI, WMI, PSI) in children, we only found significant nonlinear associations between CRP and PSI (*P* for nonlinearity = 0.011) (Additional file 1: Fig. S3).

### Sensitivity analyses

For sensitivity analysis 1, we found that index 2 was significantly and negatively correlated with both the FSIQ and each sub-dimension (VCI, VSI, FRI, WMI, PSI) (Additional file 1: Table S9). After adjusting for confounders and performing multiple comparisons for correction, in addition to the results of the main model remaining stable, we further found that IL-6 was negatively associated with VCI and WMI, and TNF-α was negatively associated with VCI and WMI (Additional file 1: Table S10). After analyses stratified by sex, we observed that IL-6 was negatively associated with FSIQ, VSI, WMI, and FRI in boys and FSIQ and VCI in girls. TNF-α had a negative correlation with WMI in boys and VCI in girls. IL-8 was found to be negatively associated with FSIQ, VSI, and WMI in boys. IFN-γ showed a negative association with WMI in boys (Additional file 1: Table S11).

For sensitivity analysis 2, stable results were observed compared to the primary analysis results (Additional file 1: Table S9-S11).

Sensitivity analyses 3 shows that when only data from Ct < 35 in the inflammatory cytokine assay were considered the results obtained remained stable after adjusting for confounding and multiple corrections (Additional file 1: Fig. S4).

## Discussion

The primary purpose of the current study was to investigate the relationship between markers of placental inflammation and children’s cognitive development. The results of the study support a possible linear association between placental immune activity and offspring cognitive development. Negative correlation between preschooler’s FSIQ and placental mRNA expression of four inflammatory cytokines (IL-8, IL-6, IFN-γ, TNF-α) was observed.

The placenta acts as the main barrier between the fetus and the mother and serves a key role in maintaining immune homeostasis at the mother–fetus interface and in utero [52]. The source of placental inflammatory cytokines may be either from the placental tissue itself or from the mother [53, 54]. Firstly, the placenta creates an immune environment that supports pregnancy, containing immune cells that promote fetoplacental growth and protect both the mother and fetus from external challenges [55]. Increased placental cytokine release may help to activate particular inflammatory pathways necessary to cause maternal insulin resistance, which is necessary for the normal development of pregnancy [56] and may also be a protective mechanism in the face of several adverse factors (trauma, hypoxia/ischemia, metabolism/obesity/diet/diabetes, stress, and pollution) [57, 58]. Numerous cytokines expressed in the placenta can play a significant role in modulating the maternal–fetal immune interface to control the mother from rejecting the fetus [59]. Secondly, maternal factors such as infection or disease are the most common causes of placental inflammation include maternal infections that activate the maternal immune system to release pro-inflammatory cytokines such as IL-6 and TNF-α, which can cross the placenta directly into fetal circulation [30]. Placental inflammation has been proven in animal models to stimulate fetal endogenous cytokine production [52, 60]. Peripheral cytokines can interact with the brain and central nervous system in several ways, such as through blood–brain barrier cells that release immunoreactive chemicals, diffusion from circumventricular organs, cytokine transporters, or cranial nerves [17]. It has been shown that cytokines and chemokines are present in human fetal forebrain cells as early as 5 weeks of gestation [61]. Although it is not clear whether cytokines in fetal brain are endogenously released, or diffusion of peripheral cytokines through blood–brain barrier, cytokines in the fetal brain may have direct effects on offspring’s later cognitive development.

We found that placental IL-6 mRNA expression was negatively associated with total cognitive scores and multiple sub-dimensional scores in preschoolers. The association remained significant at the FSIQ and FRI, even after multiple corrections were made. When further adjusted for cognitive test age, IL-6 was found to present in negative correlation with FSIQ, VCI, FRI, and WMI. Studies have shown that the pro-inflammatory cytokine IL-6 has the ability to penetrate both the placental and blood–brain barriers [62, 63]. Elevated levels of maternal IL-6 have been shown to trigger inflammatory processes in the fetal brain [64] through direct placental transfer to the fetal compartment [65] or indirectly via placental inflammation [60]. According to a longitudinal study conducted at the University of California, there exists a negative correlation between maternal IL-6 concentrations during pregnancy and FRI in children [42], which further identified pars triangularis volume as an important mediator of the relationship between maternal IL-6 concentrations and offspring FRI [42]. Other studies by the group also demonstrated that maternal IL-6 concentrations were negatively associated with working memory performance and impulse control at age 2 years, as well as cognitive ability in offspring at 12 years of age [66–68]. Other epidemiological studies have also suggested that increased maternal IL-6 concentrations are correlated with poorer cognitive ability in infants [27]. Thus, the effects of IL-6 on cognitive development are not only implicated in working memory and fluid reasoning but may also have important implications for verbal comprehension.

IL-8 is a well-known circulating inflammatory cytokine and a mediator of the systemic inflammatory response that plays multiple roles in the brain, including roles in neurogenesis, synaptic plasticity, and neurotransmission [69, 70]. Several epidemiological studies have found that increased maternal prenatal IL-8 is related to altered brain structure and elevated risk of psychiatric symptoms in offspring [71–73]. Dozmorov et al. [41] found that maternal prenatal plasma IL-8 was negatively associated with child spatial abilities but positively associated with verbal abilities. Some studies also suggest that IL-8 also has positive effects on child development. For example, higher child self-regulation is found to be associated with the concentration of IL-8 in maternal serum [74]. Also, elevated gestational levels of IL-8 were linked with enhanced performance on both the Drawing task and Tactile Finger Recognition Task [18]. Gilman found that reduced maternal IL-8 was correlated with the occurrence of neurologic abnormalities [75]. In the current study, IL-8 was found to be strongly correlated with FSIQ and FRI, even after multiple corrected comparisons. It was inconsistent with the current study, as probably because the neuroprotective or neurotoxic effects of IL-8 in the brain depend on the levels, conditioning, and co-occurrence of other cells or molecules.

Meanwhile, placental TNF-α and IFN-γ mRNA expression was found to be negatively associated with preschool children’s FSIQ. Current epidemiological studies also support that these two cytokines may play an important role in children’s cognitive development, whether during pregnancy or at birth or in childhood [16, 18, 27, 76]. The Collaborative Perinatal Project study found that children with higher maternal TNF-α levels at the second and third trimesters of pregnancy had lower IQ, reduced cognitive performance, and increased problem scores at age 7 years [18]. Another prospective cohort study found that IFN-γ in cord blood was associated with a reduced risk of having a low performance intelligence quotient at age 5 years and that TNF-a was protective against low verbal intelligence quotient in preterm infants [76]. Rhea study finds that children with high serum TNF-α and IFN-γ have decreased score on memory performance at preschool age [16]. A study based on children with autism showed that IFN-γ levels in children were negatively associated with WMI [77]. After further adjusted for children’s age at cognitive testing, we similarly found that TNF-α was negatively both VCI and WMI, as well as IFN-γ was negatively correlated with WMI, which supports the important role of these two cytokines in memory development or verbal comprehension in children.

We did not find significant relationships between IL-1β, CRP, IL-4, and IL-10 and preschoolers’ cognitive functions after multiple corrected comparisons. Some epidemiological studies likewise did not find any association [16, 18, 27, 41, 76, 77]. However, Dozmorov et al. [41] found that high maternal plasma IL-1β in the first two trimesters of pregnancy was associated with low general conceptual, nonverbal, and spatial ability. Nazzari et al. [78] found that serum CRP levels at 30–33 weeks of gestation were associated with low infant cognitive performance. Krakowiak et al. [79] found that neonatal IL-4 was associated with increased odds of mild/moderate autism ASD and that high IL-4 was negatively related to nonverbal cognitive abilities in male subjects with ASD. In a cohort study of preterm infants, top quartile concentrations of IL-4 in the first month of life were associated with an elevated risk of low IQ and low processing speed at age 10 years [80]. More prospective studies are necessary to support evidence on the association of maternal prenatal IL-1β, CRP, and IL-4 levels with offspring’s cognitive development and the possible sensitivity period of the effect. It should be noted that the transcripts corresponding to the primers for IL-10 in this study contain both coding (NM_001382624.1, NM_000572.3) and non-coding RNAs (NR_168466.1), and non-coding transcripts have the potential to have a significant impact on many facets of RNA biology, including splicing and modification. Although this study did not find a significant correlation between children’s cognitive development and the transcript levels of IL-10, the association with each of the transcripts of IL-10 as well as the protein expression need to be further explored.

The inflammatory response is a complex physiological process involving the interaction of multiple inflammatory cells, molecules, and pathways. Armstrong-Wells et al. [11] found that placental inflammation on the fetal side was associated with increased maternal IL-6 and IL-8 at delivery, as well as increased fetal IL-6, IL-8, and TNF-α. They suggested that IL-8 and IL-6—with funisitis—appear to be particularly important in the fetal inflammatory response and children’s neurological outcome [11]. Based on the function of inflammatory cytokines in the inflammatory response, we exploratively fitted the integrated anti-inflammatory index (index 1) and integrated pro-inflammatory index (index 2). After adjusting for potential confounders and children’s age at cognitive testing, we found that the index of integrated six typical pro-inflammatory cytokines (IL-8, IL-6, TNF-α, IFN-γ, IL-1β, CRP) was negatively associated with all cognitive developmental dimensions of WPPSI, and this association was stronger than for individual cytokines. This may be due to the synergistic effects among inflammatory cytokines on cognitive function. The findings would highlight the importance of comprehensive assessment of multiple classes of cytokines and the possible pathways in influencing individual’s cognition.

During inflammation process, both mRNA expression and protein expression of inflammatory cytokines change, and the expression levels of transcriptional biomarkers can indirectly reflect protein expression to some extent. However, it should be acknowledged that the association between mRNA and protein expression levels of inflammatory cytokines is not invariable and may be influenced by various factors, such as post-translational modifications that lead to translation inefficiency. However, mRNA assays are more feasible than protein levels in large-sample, multicytokine assays. In addition, at the transcriptional level, differences based on sex have been observed in the human placenta, involving genes that are associated with placental development, maintenance of pregnancy, and maternal immune tolerance for the fetus [81]. After stratifying by sex, we observed that IL-8 and IFN-γ were negatively associated with cognitive scores mainly in boys. The pro-inflammatory index similarly showed a significant association with cognitive scores (FSIQ, WMI, PSI) in boys, suggesting that there may be sex differences in the effects of different inflammatory cytokines on children’s cognitive development. The findings of the present study further add to the evidence that sex-specific susceptibility to poorer neurodevelopmental outcomes originates in the fetus. Existing studies similarly support the important role of the placenta as an organ in producing a sexually dimorphic response to intrauterine pressure exposure [82, 83]. Trophoblast cells of placenta or chorion from male fetuses in pregnancy produced higher levels of TNF-α after LPS stimulation, and produced lower levels of IL-10 and G-CSF than those from female fetuses, suggesting that in the presence of a male fetus, trophoblast cells have likely to produce a more pro-inflammatory environment [84]. It should be clarified that the internal reference gene 18S selected for this study covers a sequence on the Y chromosome that was updated for the latest study. However, by comparing the Ct amplified by 18S, we found that the Ct was essentially the same for males and females, suggesting that the amplification of this primer sequence is stable between the sexes (Additional file 1: Table S12).

Results from restricted cubic spline model demonstrated a similar linear or monotonic association of inflammatory cytokine mRNA expression (IL-6, IL-8, TNF-α, IFN-γ) with cognitive development levels, suggesting that this association exhibits a relatively stable dose–response relationship. It is important to note that although this study was set up with 45 cycles of the real-time PCR, test wells with a Ct > 40 were disregarded (per MIQE guidelines) [85]. Existing studies also support the use of 40 or even 45 cycles in qPCR assays in human placental samples [49–51, 86]. Meanwhile, we separately analyzed the associations of samples with Ct < 35 for IL-1β, IL-8, IL-6, TNF-α, CRP, and IL-4 with cognitive development in children and found that the results were consistent with those of the main analyses. We note that of the eight inflammatory cytokines only IFN-γ had a mean Ct > 35, which also suggests that the mRNA expression corresponding to this target gene was low overall in the placental samples studied. During early pregnancy, IFN-γ production is mainly limited to chorionic villous [87], but both IFN-γR1 and IFN-γR2 proteins are expressed in placental trophoblasts throughout human pregnancy [88]. Once the pregnancy is established and enters the second and third trimesters, IFN-γ production decreases; Thus, reduced IFN-γ secretion by late placental trophoblasts is physiologically required to avoid uncontrolled invasion [88].

Placental inflammation may mediate the effects of prenatal adverse pregnancy environment on cognitive development. Maternal environment during pregnancy such as infection, metabolic dysfunction, high maternal BMI, family economic status, and alcohol consumption can have potential effects on the developing fetal brain and long-term cognition [89–93]. These prenatal adverse conditions affect the function of the placenta in regulating nutrient transport, endocrine function, and immune tolerance [94, 95] and thus its involvement in fetal growth restriction, hypoxia, and associated cognitive and brain development [96–99]. It was found that the relationship between maternal obesity and offspring visuomotor ability may be influenced in part by maternal inflammation [100]. The current study has adjusted these important factors to reduce the potential confounding effect of the association of placental inflammatory factors with cognitive development.

It is important to recognize that there exist certain limitations that need to be acknowledged. Firstly, we only assayed placental cytokines at the mRNA expression and did not validate them at the level of corresponding protein expression. The results found in this study may not be fully applicable directly to proteomics. Second, the placental samples tested in the present study included both maternal and fetal surfaces and could not further explore whether the association of inflammatory burden on different surfaces of the placenta with cognitive development in children differs and the possible mechanisms of bidirectional transfer. Third, although our participants did not include pregnant women with mental disorders, we did not collect the detailed information on children’s family history of neurodevelopmental disorders. However, it was shown that the point prevalence of mental disorders in China was 1.1% in 2013 [101]. Fourth, although rigorous quality control and FDR correction for multiple testing were implemented, there is still the possibility of spurious association estimates due to possible measurement errors introduced by not performing repeated measurements of qPCR. We encourage future studies to perform more stringent quality control and to perform at least two repeated measurements to validate our conclusions. Fifth, this present study is only an exploratory study conducted in China. We used WPPSI to assess children’s cognitive development. The findings would be further validated by different cognitive assessment instruments and repeated tests at later multiple ages are encouraged to be performed to observe the long-lasting effect of placental cytokines. In addition, there are some drivers in early childhood that may influence cognitive development have not been collected, such as in-home nurturance, stimulation, peer contact, the quality and duration of preschool and kindergarten programs. Therefore, we cannot further explore the potential modifying effects of these important early childhood covariates.

## Conclusions

In conclusion, this prospective study suggests that placental pro-inflammatory milieu may have long-term effects on children’s cognitive development. High mRNA expression of pro-inflammatory cytokines (IL-8, IL-6, TNF-α, IFN-γ) may be early predictive markers of low cognitive function in children in utero. Future studies are needed to validate our findings.

### Supplementary Information

**Additional file 1: Fig. S1. **Directed acyclic graph of the relationship between placental cytokine mRNA expression and children’s cognitive development. **Fig. S2. **Spearman correlation coefficients between placental inflammatory cytokines.** Fig. S3. **Restricted cubic spline analysis of the association between ln conversion of placental inflammatory cytokine mRNA expression (IL-8, IL-1β, IL-6, TNF-α, CRP, IFN-γ, IL-10 and IL-4) and children’s cognitive scores (VCI, VSI, FRI, WMI, PSI and FSIQ) (adjusted for maternal age, maternal IQ, family monthly income per capita, pre-pregnancy BMI, parity, maternal metabolic dysfunctions, maternal fever during pregnancy, maternal infection or inflammation conditions during pregnancy, maternal alcohol use during pregnancy, father’s education level, children’s sex, and placental efficiency.). **Fig. S4. **The associations between placental inflammatory cytokines mRNA expression (IL-8, IL-1β, IL-6, TNF-α, CRP, IL-10 and IL-4) and children’s cognitive performance (VCI, VSI, FRI, WMI, PSI and FSIQ) by linear regression model when considering only data with Ct<35 in the mRNA assay.** Table S1. **Sequences of the oligonucleotides utilized in RT-qPCR. **Table S2. **RT-qPCR quality control measures.** Table S3. **Detailed information on confounders.** Table S4. **Comparisons of the basic demographic characteristics of the included and excluded populations. **Table S5. **Ct of internal reference genes and target genes in placental qPCR assays in the included population.** Table S6. **Comparison of cognitive scores in children with and without placental data. **Table S7. **The association between placental eight cytokines mRNA expression and children’s cognitive performance in a model by multiple linear regression model. **Table S8.** The association between interaction of placental inflammatory cytokines mRNA expression*sex and children’s cognitive performance. **Table S9. **Sensitivity analysis of the association between placental summary index of cytokines and children’s cognitive performance by multivariate linear regression analyses. **Table S10. **Sensitivity analysis of the association between placental each inflammatory cytokine mRNA expression and children’s cognitive performance by multivariate linear regression models. **Table S11. **Sensitivity analysis of the association between placental cytokine mRNA expression and children’s cognitive performance by sex.** Table S12. **Comparison of Ct of endogenous reference RNA-18S between sexes.

**Additional file 2. **STROBE checklist.

## Data Availability

The datasets used and/or analyzed in the current study are available upon reasonable request to the corresponding author.

## Abbreviations

CRP: C-reactive protein
DAG: Directed acyclic graph
FRI: Fluid reasoning index
FSIQ: Full-scale intelligence quotient
IFN-γ: Interferon-γ
IL: Interleukin
IQ: Intelligence quotient
MABC: The Ma’anshan birth cohort
PSI: Processing speed index
RT-qPCR: Real-time quantitative polymerase chain reaction
SD: Standard deviation
TNF-α: Tumor necrosis factor-α
VCI: Verbal comprehension index
VSI: Visual spatial index
WMI: Working memory index
WPPSI-IV: The Wechsler Preschool and Primary Scale of Intelligence, Fourth Edition

## References

[1] Zhou H, Ye R, Sylvia S, Rose N, Rozelle S (2020). "At three years of age, we can see the future": cognitive skills and the life cycle of rural Chinese children. Demogr Res.

[2] Leech SL, Larkby CA, Day R, Day NL (2006). Predictors and correlates of high levels of depression and anxiety symptoms among children at age 10. J Am Acad Child Adolesc Psychiatry.

[3] Biermann J, Franze M, Hoffmann W (2020). Social developmental delays among 3 to 6 year old children in preschools in German social hotspots: results of a dynamic prospective cohort study. BMC Pediatr.

[4] Hung GC, Pietras SA, Carliner H, Martin L, Seidman LJ, Buka SL (2016). Cognitive ability in childhood and the chronicity and suicidality of depression. Br J Psychiatry.

[5] Guttmacher AE, Maddox YT, Spong CY (2014). The Human Placenta Project: placental structure, development, and function in real time. Placenta.

[6] Lester BM, Marsit CJ (2018). Epigenetic mechanisms in the placenta related to infant neurodevelopment. Epigenomics.

[7] Kim CJ, Romero R, Chaemsaithong P, Kim JS (2015). Chronic inflammation of the placenta: definition, classification, pathogenesis, and clinical significance. Am J Obstet Gynecol.

[8] Mattuizzi A, Sauvestre F, André G, Poingt M, Camberlein C, Carles D (2020). Adverse perinatal outcomes of chronic intervillositis of unknown etiology: an observational retrospective study of 122 cases. Sci Rep.

[9] Zhang Q, Lu HY, Wang JX, Mao XQ, Ma JL, Lu JY (2015). Relationship between placental inflammation and fetal inflammatory response syndrome and brain injury in preterm infants. Zhongguo Dang Dai Er Ke Za Zhi.

[10] Liao Y, Zhang YN, Liu XL, Lu YY, Zhang LL, Xi T (2018). Maternal murine cytomegalovirus infection during pregnancy up-regulates the gene expression of toll-like receptor 2 and 4 in placenta. Curr Med Sci.

[11] Armstrong-Wells J, Donnelly M, Post MD, Manco-Johnson MJ, Winn VD, Sébire G (2015). Inflammatory predictors of neurologic disability after preterm premature rupture of membranes. Am J Obstet Gynecol.

[12] Korzeniewski SJ, Romero R, Cortez J, Pappas A, Schwartz AG, Kim CJ (2014). A "multi-hit" model of neonatal white matter injury: cumulative contributions of chronic placental inflammation, acute fetal inflammation and postnatal inflammatory events. J Perinat Med.

[13] Rosenfeld CS (2021). The placenta-brain-axis. J Neurosci Res.

[14] Hope S, Hoseth E, Dieset I, Mørch RH, Aas M, Aukrust P (2015). Inflammatory markers are associated with general cognitive abilities in schizophrenia and bipolar disorder patients and healthy controls. Schizophr Res.

[15] Kogan S, Ospina LH, Mittal VA, Kimhy D (2020). The impact of inflammation on neurocognition and risk for psychosis: a critical review. Eur Arch Psychiatry Clin Neurosci.

[16] Kyriklaki A, Margetaki K, Kampouri M, Koutra K, Bitsios P, Chalkiadaki G (2019). Association between high levels of inflammatory markers and cognitive outcomes at 4 years of age: the Rhea mother-child cohort study, Crete. Greece Cytokine.

[17] Jiang NM, Cowan M, Moonah SN, Petri WA (2018). The impact of systemic inflammation on neurodevelopment. Trends Mol Med.

[18] Ghassabian A, Albert PS, Hornig M, Yeung E, Cherkerzian S, Goldstein RB (2018). Gestational cytokine concentrations and neurocognitive development at 7 years. Transl Psychiatry.

[19] Esteban-Cornejo I, Martinez-Gomez D, Gómez-Martínez S, Del Campo-Vecino J, Fernández-Santos J, Castro-Piñero J (2016). Inflammatory biomarkers and academic performance in youth. The UP & DOWN Study. Brain Behav Immun..

[20] Gall AR, Amoah S, Kitase Y, Jantzie LL (2022). Placental mediated mechanisms of perinatal brain injury: evolving inflammation and exosomes. Exp Neurol.

[21] Knuesel I, Chicha L, Britschgi M, Schobel SA, Bodmer M, Hellings JA (2014). Maternal immune activation and abnormal brain development across CNS disorders. Nat Rev Neurol.

[22] Estes ML, McAllister AK (2016). Maternal immune activation: implications for neuropsychiatric disorders. Science.

[23] Deverman BE, Patterson PH (2009). Cytokines and CNS development. Neuron.

[24] Dammann O, O'Shea TM (2008). Cytokines and perinatal brain damage. Clin Perinatol.

[25] McAfoose J, Baune BT (2009). Evidence for a cytokine model of cognitive function. Neurosci Biobehav Rev.

[26] Meyer U, Feldon J, Yee BK (2009). A review of the fetal brain cytokine imbalance hypothesis of schizophrenia. Schizophr Bull.

[27] Camerota M, Wylie AC, Goldblum J, Wideman L, Cheatham CL, Propper CB (2022). Testing a cascade model linking prenatal inflammation to child executive function. Behav Brain Res.

[28] Bodnar TS, Raineki C, Wertelecki W, Yevtushok L, Plotka L, Zymak-Zakutnya N (2018). Altered maternal immune networks are associated with adverse child neurodevelopment: impact of alcohol consumption during pregnancy. Brain Behav Immun.

[29] Majerczyk D, Ayad EG, Brewton KL, Saing P, Hart PC. Systemic maternal inflammation promotes ASD via IL-6 and IFN-γ. Biosci Rep. 2022;42(11):BSR20220713. 10.1042/BSR20220713 PMC9670245 36300375

[30] Zawadzka A, Cieślik M, Adamczyk A. The role of maternal immune activation in the pathogenesis of autism: a review of the evidence, proposed mechanisms and implications for treatment. Int J Mol Sci. 2021;22(21):11516. 10.3390/ijms222111516 PMC8584025 34768946

[31] Kelly RS, Lee-Sarwar K, Chen YC, Laranjo N, Fichorova R, Chu SH, et al. Maternal inflammatory biomarkers during pregnancy and early life neurodevelopment in offspring: results from the VDAART study. Int J Mol Sci. 2022;23(23):15249. 10.3390/ijms232315249 PMC9739845 36499584

[32] Galera C, Barbosa S, Collet O, Khalfallah O, Aouizerate B, Sutter-Dalley AL (2021). Cord serum cytokines at birth and children's anxiety-depression trajectories from 3 to 8 years: the EDEN mother-child cohort. Biol Psychiatry.

[33] Voltas N, Arija V, Hernández-Martínez C, Jiménez-Feijoo R, Ferré N, Canals J (2017). Are there early inflammatory biomarkers that affect neurodevelopment in infancy?. J Neuroimmunol.

[34] Nelson KB, Blair E (2011). The placenta and neurologic and psychiatric outcomes in the child: study design matters. Placenta.

[35] Trenova AG, Slavov GS, Manova MG, Miteva LD, Stanilova SA (2018). A role of cytokine gene polymorphisms in cognitive functioning. Folia Med (Plovdiv).

[36] Ratnayake U, Quinn T, Walker DW, Dickinson H (2013). Cytokines and the neurodevelopmental basis of mental illness. Front Neurosci.

[37] Meyyazhagan A, Kuchi Bhotla H, Pappuswamy M, Tsibizova V, Al Qasem M, Di Renzo GC (2023). Cytokine see-saw across pregnancy, its related complexities and consequences. Int J Gynaecol Obstet.

[38] Zhou J, Teng Y, Zhang F, Ru X, Li P, Wang J (2022). Sex-specific association between placental inflammatory cytokine mRNA expression and preschoolers' behavioral development: the Ma'anshan birth cohort study. Brain Behav Immun.

[39] Wechsler D (2012). Wechsler preschool and primary scale of intelligence—fourth edition.

[40] Zhu YD, Wu XY, Yan SQ, Huang K, Tong J, Gao H (2020). Domain- and trimester-specific effect of prenatal phthalate exposure on preschooler cognitive development in the Ma'anshan Birth Cohort (MABC) study. Environ Int.

[41] Dozmorov MG, Bilbo SD, Kollins SH, Zucker N, Do EK, Schechter JC (2018). Associations between maternal cytokine levels during gestation and measures of child cognitive abilities and executive functioning. Brain Behav Immun.

[42] Rasmussen JM, Graham AM, Gyllenhammer LE, Entringer S, Chow DS, O'Connor TG (2022). Neuroanatomical correlates underlying the association between maternal interleukin 6 concentration during pregnancy and offspring fluid reasoning performance in early childhood. Biol Psychiatry Cogn Neurosci Neuroimaging.

[43] Tennant PWG, Murray EJ, Arnold KF, Berrie L, Fox MP, Gadd SC (2021). Use of directed acyclic graphs (DAGs) to identify confounders in applied health research: review and recommendations. Int J Epidemiol.

[44] Pike N (2011). Using false discovery rates for multiple comparisons in ecology and evolution. Methods Ecol Evol.

[45] Koh K. Maternal breastfeeding and children’s cognitive development. Soc Sci Med. 2017;187:101–8. 10.1016/j.socscimed.2017.06.012 28672220

[46] Jirout J, LoCasale-Crouch J, Turnbull K, Gu Y, Cubides M, Garzione S, et al. How Lifestyle Factors Affect Cognitive and Executive Function and the Ability to Learn in Children. Nutrients. 2019;11(8):1953. 10.3390/nu11081953 PMC6723730 31434251

[47] Radesky JS, Christakis DA (2016). Increased screen time: implications for early childhood development and behavior. Pediatr Clin North Am.

[48] Tanskanen AO, Danielsbacka M (2018). Multigenerational effects on children's cognitive and socioemotional outcomes: a within-child investigation. Child Dev.

[49] Huang X, Baumann M, Nikitina L, Wenger F, Surbek D, Körner M (2013). RNA degradation differentially affects quantitative mRNA measurements of endogenous reference genes in human placenta. Placenta.

[50] Adibi JJ, Whyatt RM, Hauser R, Bhat HK, Davis BJ, Calafat AM (2010). Transcriptional biomarkers of steroidogenesis and trophoblast differentiation in the placenta in relation to prenatal phthalate exposure. Environ Health Perspect.

[51] Amu S, Hahn-Zoric M, Malik A, Ashraf R, Zaman S, Kjellmer I (2006). Cytokines in the placenta of Pakistani newborns with and without intrauterine growth retardation. Pediatr Res.

[52] Howerton CL, Bale TL (2012). Prenatal programing: at the intersection of maternal stress and immune activation. Horm Behav.

[53] Zhu MJ, Du M, Nathanielsz PW, Ford SP (2010). Maternal obesity up-regulates inflammatory signaling pathways and enhances cytokine expression in the mid-gestation sheep placenta. Placenta.

[54] Saito S (2000). Cytokine network at the feto-maternal interface. J Reprod Immunol.

[55] Racicot K, Kwon JY, Aldo P, Silasi M, Mor G (2014). Understanding the complexity of the immune system during pregnancy. Am J Reprod Immunol.

[56] Redman CW, Sargent IL (2003). Pre-eclampsia, the placenta and the maternal systemic inflammatory response–a review. Placenta.

[57] Parkington HC, Sheehan PM, Coleman HA, Brennecke SP (2020). NO placental inflammation. J Physiol.

[58] Wright RJ, Visness CM, Calatroni A, Grayson MH, Gold DR, Sandel MT (2010). Prenatal maternal stress and cord blood innate and adaptive cytokine responses in an inner-city cohort. Am J Respir Crit Care Med.

[59] Dealtry GB, O'Farrell MK, Fernandez N (2000). The Th2 cytokine environment of the placenta. Int Arch Allergy Immunol.

[60] Hsiao EY, Patterson PH (2012). Placental regulation of maternal-fetal interactions and brain development. Dev Neurobiol.

[61] Mousa A (1999). SeigerA, Kjaeldgaard A, Bakhiet M: Human first trimester forebrain cells express genes for inflammatory and anti-inflammatory cytokines. Cytokine.

[62] Zaretsky MV, Alexander JM, Byrd W, Bawdon RE (2004). Transfer of inflammatory cytokines across the placenta. Obstet Gynecol.

[63] Banks WA, Kastin AJ, Gutierrez EG (1994). Penetration of interleukin-6 across the murine blood-brain barrier. Neurosci Lett.

[64] Wu WL, Hsiao EY, Yan Z, Mazmanian SK, Patterson PH (2017). The placental interleukin-6 signaling controls fetal brain development and behavior. Brain Behav Immun.

[65] Dahlgren J, Samuelsson AM, Jansson T, Holmäng A (2006). Interleukin-6 in the maternal circulation reaches the rat fetus in mid-gestation. Pediatr Res.

[66] Rudolph MD, Graham AM, Feczko E, Miranda-Dominguez O, Rasmussen JM, Nardos R (2018). Maternal IL-6 during pregnancy can be estimated from newborn brain connectivity and predicts future working memory in offspring. Nat Neurosci.

[67] Rasmussen JM, Graham AM, Entringer S, Gilmore JH, Styner M, Fair DA (2019). Maternal Interleukin-6 concentration during pregnancy is associated with variation in frontolimbic white matter and cognitive development in early life. Neuroimage.

[68] Graham AM, Rasmussen JM, Rudolph MD, Heim CM, Gilmore JH, Styner M (2018). Maternal systemic interleukin-6 during pregnancy is associated with newborn amygdala phenotypes and subsequent behavior at 2 years of age. Biol Psychiatry.

[69] Tsai SJ (2021). Role of interleukin 8 in depression and other psychiatric disorders. Prog Neuropsychopharmacol Biol Psychiatry.

[70] Baggiolini M, Clark-Lewis I (1992). Interleukin-8, a chemotactic and inflammatory cytokine. FEBS Lett.

[71] Mac Giollabhui N, Breen EC, Murphy SK, Maxwell SD, Cohn BA, Krigbaum NY (2019). Maternal inflammation during pregnancy and offspring psychiatric symptoms in childhood: timing and sex matter. J Psychiatr Res.

[72] Ellman LM, Deicken RF, Vinogradov S, Kremen WS, Poole JH, Kern DM (2010). Structural brain alterations in schizophrenia following fetal exposure to the inflammatory cytokine interleukin-8. Schizophr Res.

[73] Brown AS, Hooton J, Schaefer CA, Zhang H, Petkova E, Babulas V (2004). Elevated maternal interleukin-8 levels and risk of schizophrenia in adult offspring. Am J Psychiatry.

[74] Yu J, Ghassabian A, Chen Z, Goldstein RB, Hornig M, Buka SL (2020). Maternal Immune activity during pregnancy and socioeconomic disparities in children's self-regulation. Brain Behav Immun.

[75] Gilman SE, Hornig M, Ghassabian A, Hahn J, Cherkerzian S, Albert PS (2017). Socioeconomic disadvantage, gestational immune activity, and neurodevelopment in early childhood. Proc Natl Acad Sci U S A.

[76] von Ehrenstein OS, Neta GI, Andrews W, Goldenberg R, Goepfert A, Zhang J (2012). Child intellectual development in relation to cytokine levels in umbilical cord blood. Am J Epidemiol.

[77] Sasayama D, Kurahashi K, Oda K, Yasaki T, Yamada Y, Sugiyama N, et al. Negative correlation between serum cytokine levels and cognitive abilities in children with autism spectrum disorder. J Intell. 2017;5(2):19. 10.3390/jintelligence5020019 PMC6526410 31162410

[78] Nazzari S, Fearon P, Rice F, Ciceri F, Molteni M, Frigerio A (2020). Neuroendocrine and immune markers of maternal stress during pregnancy and infant cognitive development. Dev Psychobiol.

[79] Krakowiak P, Goines PE, Tancredi DJ, Ashwood P, Hansen RL, Hertz-Picciotto I (2017). Neonatal cytokine profiles associated with autism spectrum disorder. Biol Psychiatry.

[80] Leviton A, Joseph RM, Allred EN, Fichorova RN, O'Shea TM, Kuban KKC (2018). The risk of neurodevelopmental disorders at age 10 years associated with blood concentrations of interleukins 4 and 10 during the first postnatal month of children born extremely preterm. Cytokine.

[81] Buckberry S, Bianco-Miotto T, Bent SJ, Dekker GA, Roberts CT (2014). Integrative transcriptome meta-analysis reveals widespread sex-biased gene expression at the human fetal-maternal interface. Mol Hum Reprod.

[82] Davis EP, Pfaff D (2014). Sexually dimorphic responses to early adversity: implications for affective problems and autism spectrum disorder. Psychoneuroendocrinology.

[83] Aiken CE, Ozanne SE (2013). Sex differences in developmental programming models. Reproduction.

[84] Challis J, Newnham J, Petraglia F, Yeganegi M, Bocking A (2013). Fetal sex and preterm birth. Placenta.

[85] Bustin SA, Benes V, Garson JA, Hellemans J, Huggett J, Kubista M (2009). The MIQE guidelines: minimum information for publication of quantitative real-time PCR experiments. Clin Chem.

[86] Adibi JJ, Hauser R, Williams PL, Whyatt RM, Thaker HM, Nelson H (2009). Placental biomarkers of phthalate effects on mRNA transcription: application in epidemiologic research. Environ Health.

[87] Paulesu L, Romagnoli R, Cintorino M, Ricci MG, Garotta G (1994). First trimester human trophoblast expresses both interferon-gamma and interferon-gamma-receptor. J Reprod Immunol.

[88] Banerjee S, Smallwood A, Moorhead J, Chambers AE, Papageorghiou A, Campbell S (2005). Placental expression of interferon-gamma (IFN-gamma) and its receptor IFN-gamma R2 fail to switch from early hypoxic to late normotensive development in preeclampsia. J Clin Endocrinol Metab.

[89] Koyama Y, Hidalgo APC, Lacey RE, White T, Jansen PW, Fujiwara T (2023). Poverty from fetal life onward and child brain morphology. Sci Rep.

[90] Cordeiro CN, Tsimis M, Burd I (2015). Infections and brain development. Obstet Gynecol Surv.

[91] Lippert RN, Brüning JC (2022). Maternal metabolic programming of the developing central nervous system: unified pathways to metabolic and psychiatric disorders. Biol Psychiatry.

[92] Graham AM, Doyle O, Tilden EL, Sullivan EL, Gustafsson HC, Marr M (2022). Effects of maternal psychological stress during pregnancy on offspring brain development: considering the role of inflammation and potential for preventive intervention. Biol Psychiatry Cogn Neurosci Neuroimaging.

[93] Norr ME, Hect JL, Lenniger CJ, Van den Heuvel M, Thomason ME (2021). An examination of maternal prenatal BMI and human fetal brain development. J Child Psychol Psychiatry.

[94] Jansson T, Powell TL (2007). Role of the placenta in fetal programming: underlying mechanisms and potential interventional approaches. Clin Sci (Lond).

[95] Fernandez-Twinn DS, Ozanne SE (2010). Early life nutrition and metabolic programming. Ann N Y Acad Sci.

[96] Vuong B, Odero G, Rozbacher S, Stevenson M, Kereliuk SM, Pereira TJ (2017). Exposure to gestational diabetes mellitus induces neuroinflammation, derangement of hippocampal neurons, and cognitive changes in rat offspring. J Neuroinflammation.

[97] Schmitz L, Kuglin R, Bae-Gartz I, Janoschek R, Appel S, Mesaros A (2018). Hippocampal insulin resistance links maternal obesity with impaired neuronal plasticity in adult offspring. Psychoneuroendocrinology.

[98] Soumiya H, Fukumitsu H, Furukawa S (2011). Prenatal immune challenge compromises development of upper-layer but not deeper-layer neurons of the mouse cerebral cortex. J Neurosci Res.

[99] Martínez-Téllez RI, Hernández-Torres E, Gamboa C, Flores G (2009). Prenatal stress alters spine density and dendritic length of nucleus accumbens and hippocampus neurons in rat offspring. Synapse.

[100] Monthé-Drèze C, Rifas-Shiman SL, Gold DR, Oken E, Sen S (2019). Maternal obesity and offspring cognition: the role of inflammation. Pediatr Res.

[101] Huang Y, Wang Y, Wang H, Liu Z, Yu X, Yan J (2019). Prevalence of mental disorders in China: a cross-sectional epidemiological study. Lancet Psychiatry.

