# Supplementary material for: Placental inflammatory cytokines mRNA expression and preschool children’s cognitive performance: a birth cohort study in China
Source: BMC Med. 2023 Nov 20;21:449. doi: 10.1186/s12916-023-03173-2 (PMC10658981; doi:10.1186/s12916-023-03173-2)
Supplement: Supplementary file 1 — Additional file 1: Fig. S1. Directed acyclic graph of the relationship between placental cytokine mRNA expression and children’s cognitive development. Fig. S2. Spearman correlation coefficients between placental inflammatory cytokines. Fig. S3. Restricted cubic spline analysis of the association between ln conversion of placental inflammatory cytokine mRNA expression (IL-8, IL-1β, IL-6, TNF-α, CRP, IFN-γ, IL-10 and IL-4) and children’s cognitive scores (VCI, VSI, FRI, WMI, PSI and FSIQ) (adjusted for maternal age, maternal IQ, family monthly income per capita, pre-pregnancy BMI, parity, maternal metabolic dysfunctions, maternal fever during pregnancy, maternal infection or inflammation conditions during pregnancy, maternal alcohol use during pregnancy, father’s education level, children’s sex, and placental efficiency.). Fig. S4. The associations between placental inflammatory cytokines mRNA expression (IL-8, IL-1β, IL-6, TNF-α, CRP, IL-10 and IL-4) and children’s cognitive performance (VCI, VSI, FRI, WMI, PSI and FSIQ) by linear regression model when considering only data with Ct<35 in the mRNA assay. Table S1. Sequences of the oligonucleotides utilized in RT-qPCR. Table S2. RT-qPCR quality control measures. Table S3. Detailed information on confounders. Table S4. Comparisons of the basic demographic characteristics of the included and excluded populations. Table S5. Ct of internal reference genes and target genes in placental qPCR assays in the included population. Table S6. Comparison of cognitive scores in children with and without placental data. Table S7. The association between placental eight cytokines mRNA expression and children’s cognitive performance in a model by multiple linear regression model. Table S8. The association between interaction of placental inflammatory cytokines mRNA expression*sex and children’s cognitive performance. Table S9. Sensitivity analysis of the association between placental summary index of cytokines and children’s cognitive [file 12916_2023_3173_MOESM1_ESM.docx]

**
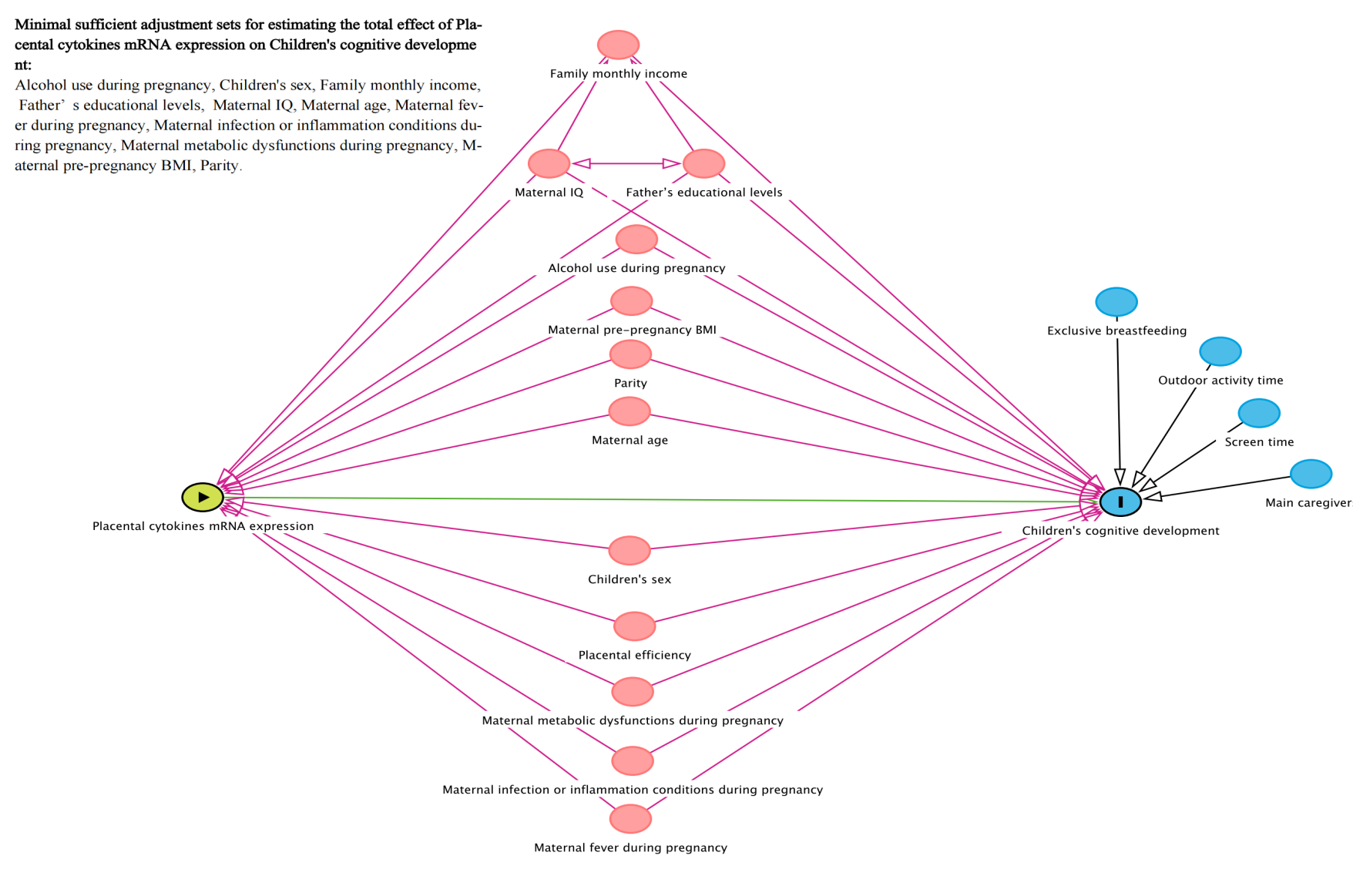
**

**Fig. S1** Directed acyclic graph of the relationship between placental cytokine mRNA expression and children’s cognitive development.


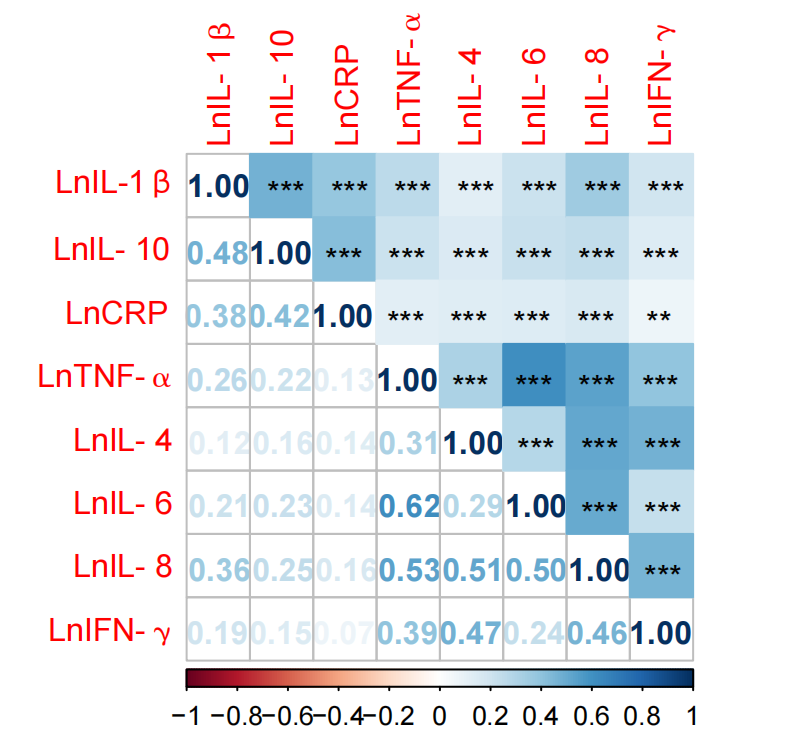


**Fig. S2** Spearman correlation coefficients between placental inflammatory cytokines.


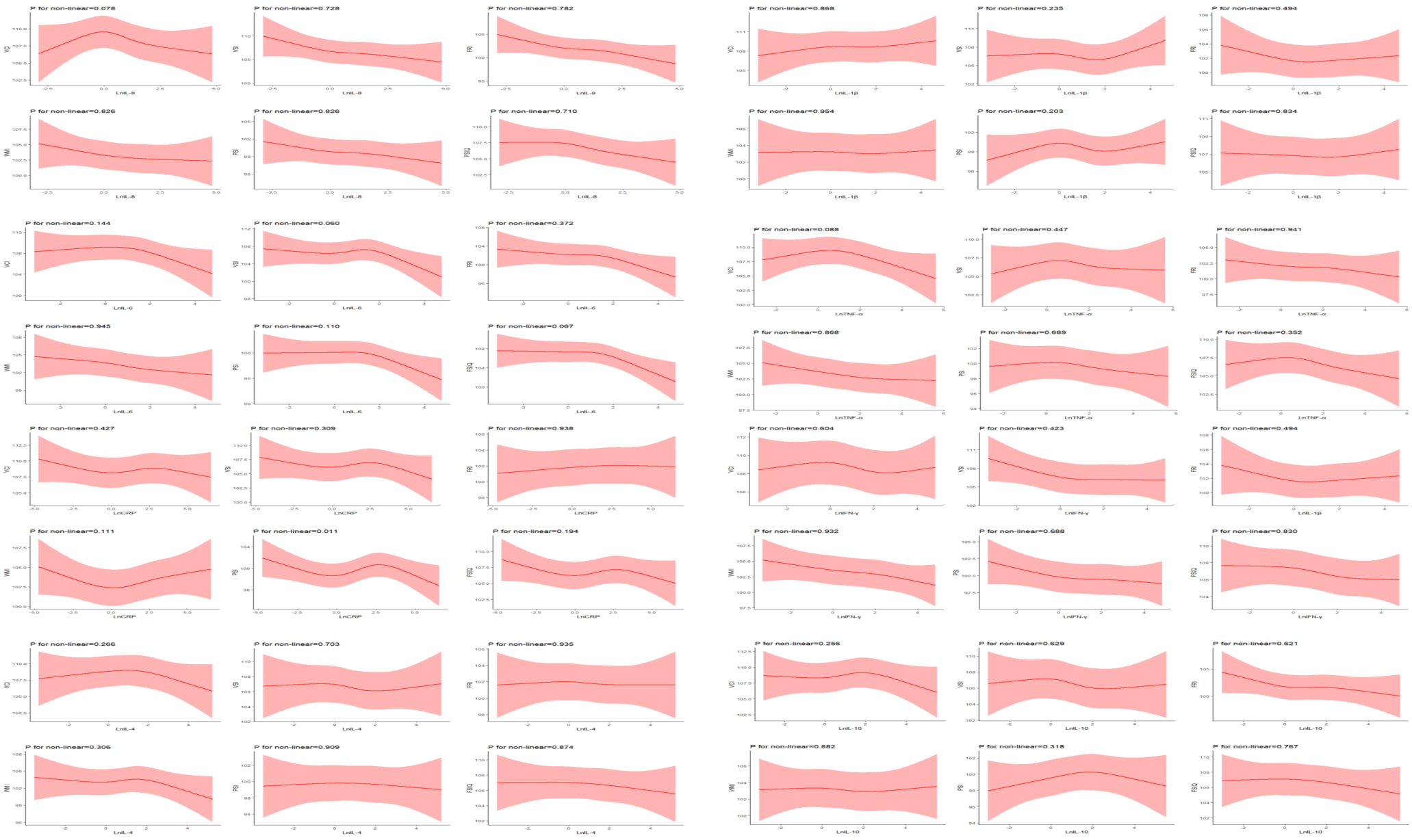


**Fig. S3** Restricted cubic spline analysis of the association between ln conversion of placental inflammatory cytokine mRNA expression (IL-8, IL-1β, IL-6, TNF-α, CRP, IFN-γ, IL-10 and IL-4) and children’s cognitive scores (VCI, VSI, FRI, WMI, PSI and FSIQ) (adjusted for maternal age, maternal IQ, family monthly income per capita, pre-pregnancy BMI, parity, maternal metabolic dysfunctions, maternal fever during pregnancy, maternal infection or inflammation conditions during pregnancy, maternal alcohol use during pregnancy, father’s education level, children’s sex, and placental efficiency.)


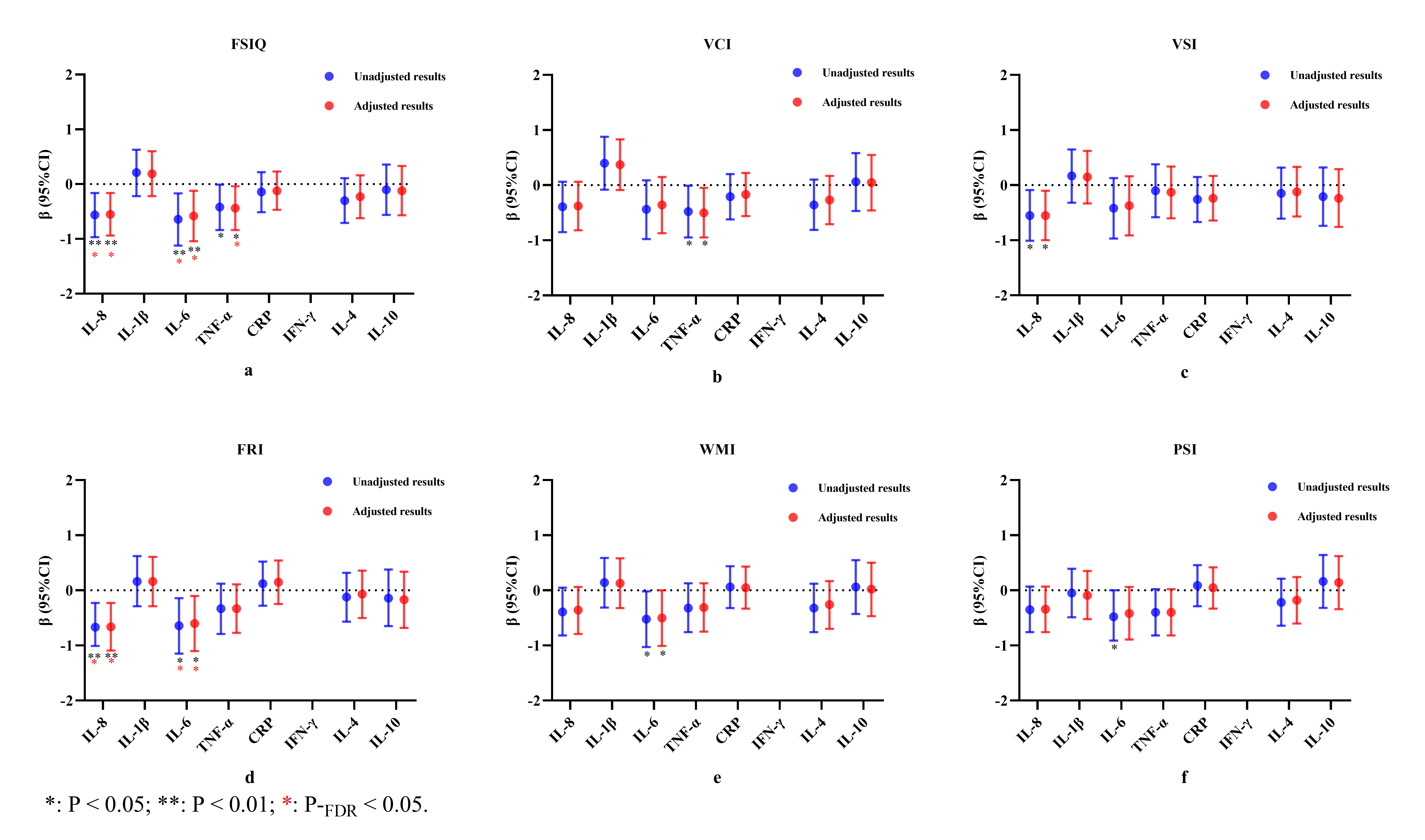


**Fig. S4** The associations between placental inflammatory cytokines mRNA expression and children’s cognitive performance by linear regression model when considering only data with Ct<35 in the mRNA assay.

**Table S1** Sequences of the oligonucleotides utilized in RT-qPCR.

| **Genes** | **Forward (5′-3′)** | **Reverse (5′-3′)** |
| --- | --- | --- |
| ***Endogenous reference RNA*** | | |
| *h-18S* | CGGCTACCACATCCAAGGAA | GCTGGAATTACCGCGGCT |
| ***Cytokine qPCR primers sequences*** | | |
| *h-IL-8* | GGCAGCCTTCCTGATTTCTG | CTTGGCAAAACTGCACCTTCA |
| *h-IL-1β* | ACAGATGAAGTGCTCCTTCCA | GTCGGAGATTCGTAGCTGGAT |
| *h-IL-6* | CAATCTGGATTCAATGAGGAGAC | CTCTGGCTTGTTCCTCACTACTC |
| *h-TNF-α* | TCTTCTCGAACCCCGAGTGA | ATGAGGTACAGGCCCTCTGA |
| *h-CRP* | CGGGCCCTTCAGTCCTAATG | AAAAGCGGGAGGTACCAGAG |
| *h-IFN-γ* | CTAATTATTCGGTAACTGACTTGA | ACAGTTCAGCCATCACTTGGA |
| *h-IL-4* | CCATGAGAAGGACACTCGCT | TCTGGTTGGCTTCCTTCACA |
| *h-IL-10* | GGTTGCCAAGCCTTGTCTGA | AGGGAGTTCACATGCGCCT |

**Table S2** RT-qPCR quality control measures.

| **Three replicate measurements** | 1. Prior to formal experiments, we firstly selected 3 random placental samples for 3 replicate measurements of all inflammatory cytokines, and we found that the coefficient of variation (CV) was almost 1%, which suggests the stability of the assay environment in this study. 2. we randomly selected 10 placental samples for 3 replicates of each gene. The mean CV was 0.70% for 18S and 1.47%, 1.19%, 1.07%, 1.47%, 1.06%, 1.14%, 0.66, and 0.95% for IL-6,IL-8, IFN-γ, TNF-α, IL-1β, CRP, IL-4, and IL-10, respectively (As shown in the table **A** on the right). Thus, the CV for both the internal reference genes and the target genes are very low. This is very close to the results of the pre-experiment before the beginning of this study, which were all around 1.0%. 3. we calculated the ∆CT of each gene with the internal reference gene for each sample based on the three replicates of the supplemented 10 samples; then, the CV of their ∆CT was calculated. The coefficients of variation are still very low (1.2%-3.39%) even after the combination of two genes (As shown in the table **B** on the right). The results show that even though despite the standardization, our results are still very robust. | A  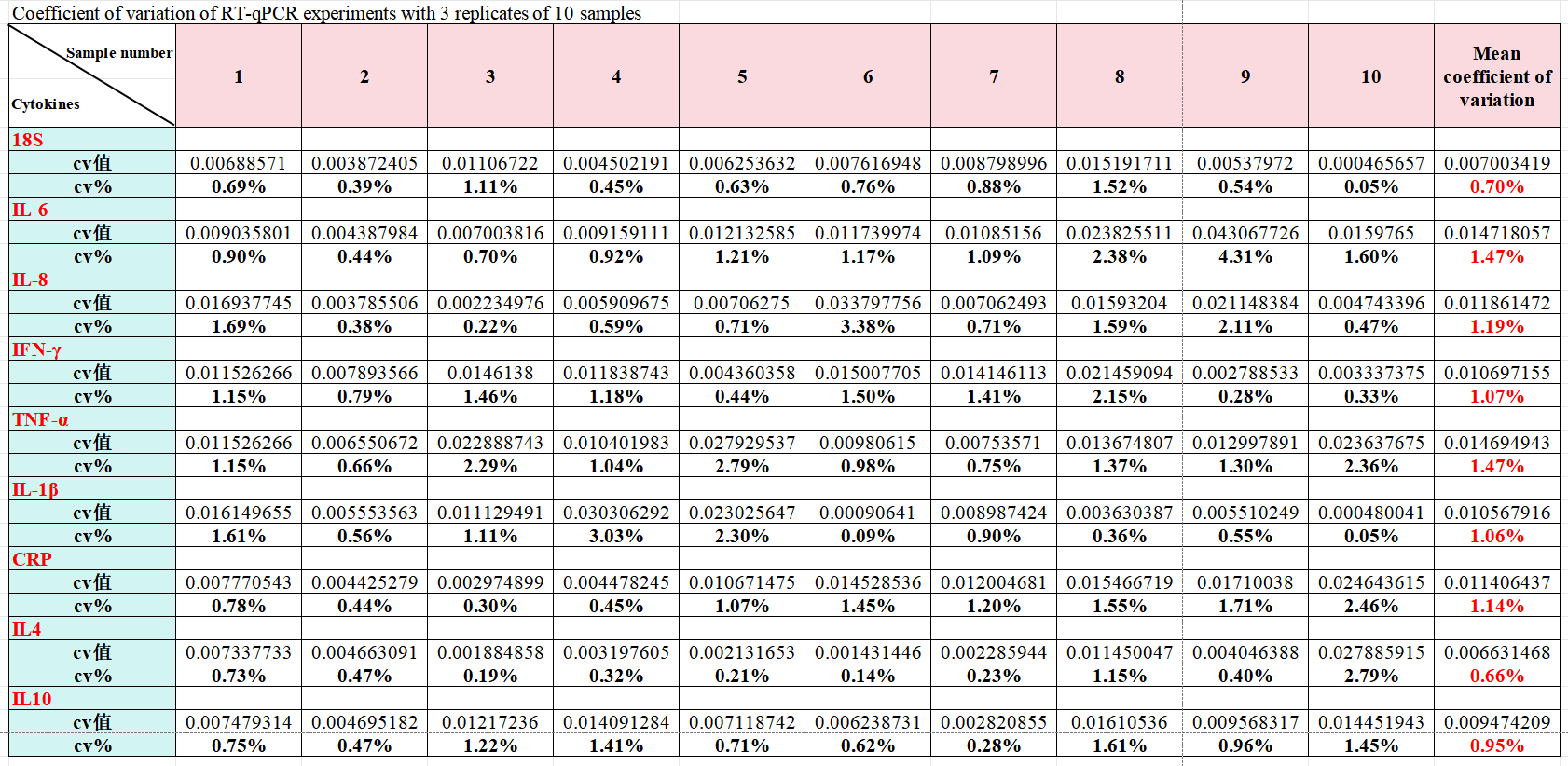  B  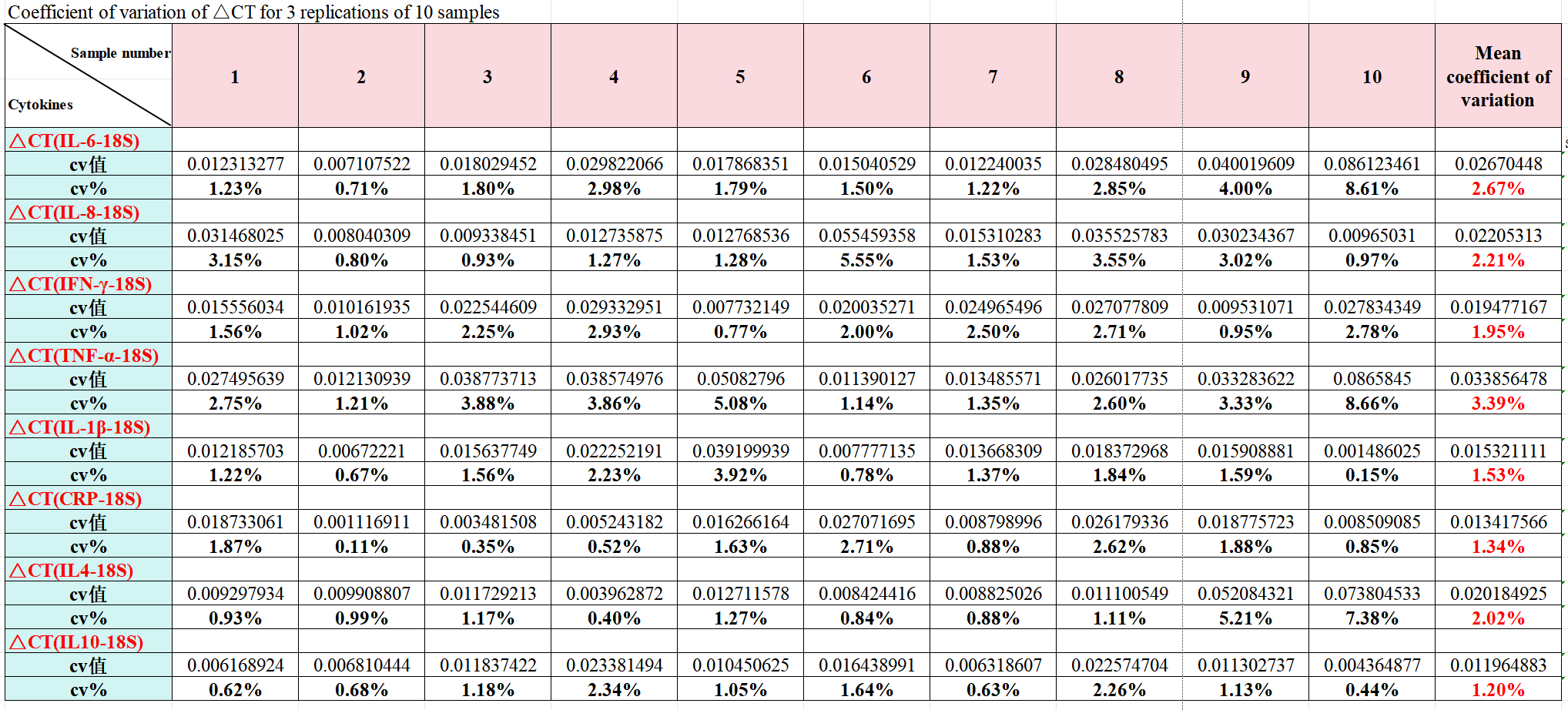 |
| --- | --- | --- |
| **Quality control (QC) samples for repeated measurements in each 96-well plate** | 1. we set up QC samples in each 96-well plate, and the QC samples were tested for the internal reference genes and each target gene. Only if the Ct values of both the internal reference and the target gene of the QC sample remained relatively stable would this set of data be considered for the measurements in this study; otherwise, the set of samples would all be re-assayed. In fact, although only one QC sample was selected in each experiment, we actually compared the data of both the internal reference and the target gene for QC. Otherwise, all samples in the group would be re-experimented. 2. Considering that one QC sample will be replaced by another when it runs out, we randomly selected 4 QC samples (a batch of tests performed in the same week using the same QC sample) and calculated their coefficients of variation. As shown in the table **C** on the right, the very low coefficients of variation (both for the internal reference genes and the target genes) indicate that the variability of the results of the QC samples from test to test is relatively small, proving the stability and reliability of our assay results. 3. Considering that the CV value of the QC samples is one kind of index that reacts to the stability of the repeated measurement results, in order to better present the stability of the assay results of the QC samples in this study, we further listed the Ct values of the QC samples and their overall linear trends. As shown in the Figure **D-H** on the right, we randomly selected four QC samples for both the internal reference gene and each target gene, and presented them with scatter plots and linear prediction models. As can be seen from the plots, the stability of our QC samples is relatively stable, both for the internal reference gene and the target genes. | **C**  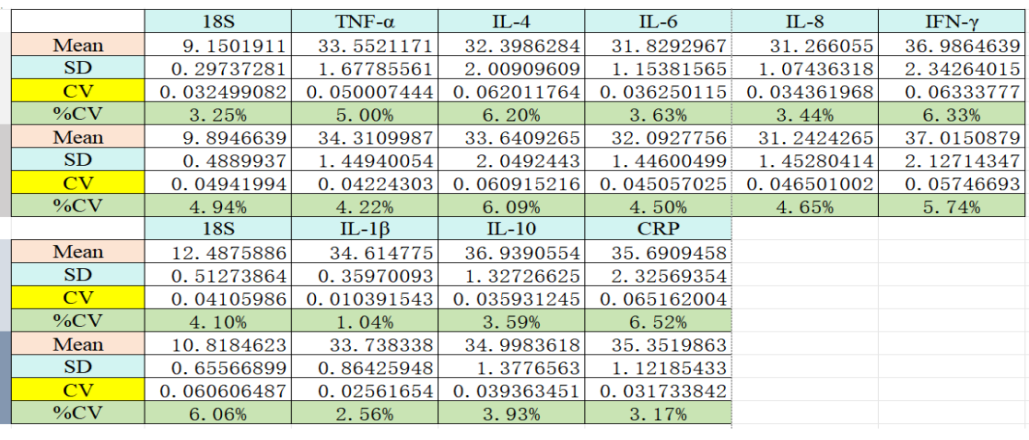  **D**  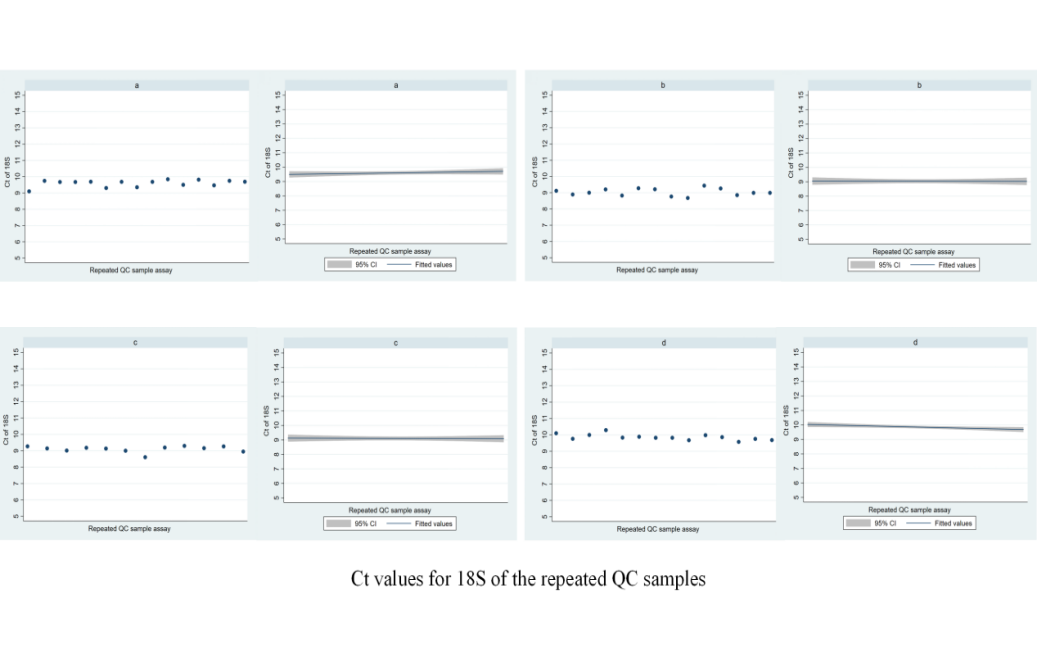  **E**  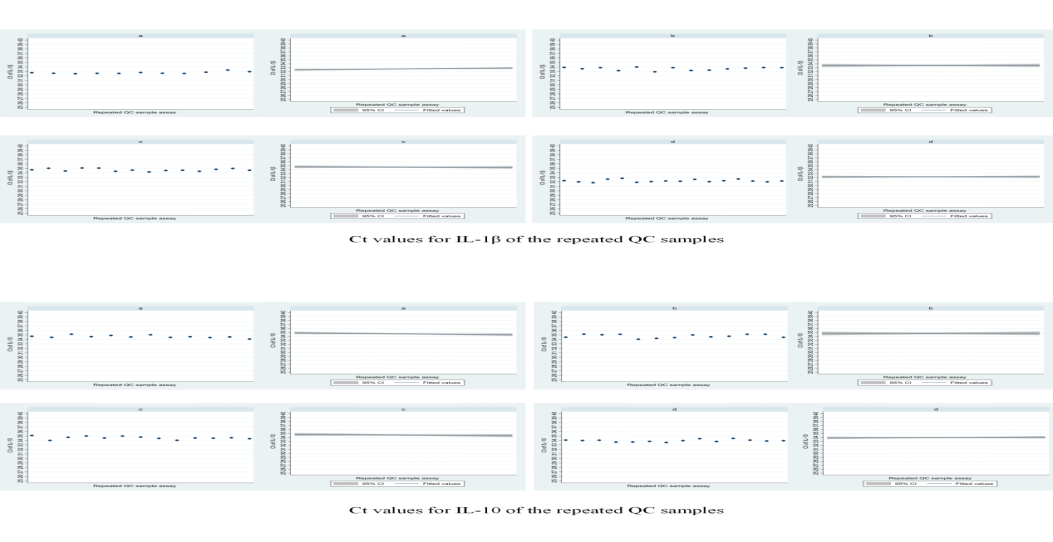  **F**  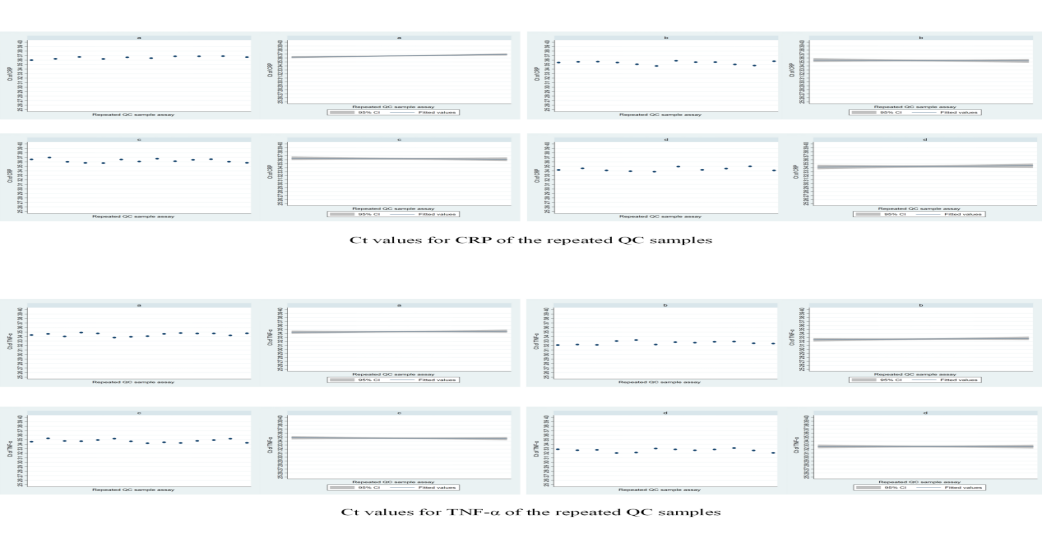  **G**  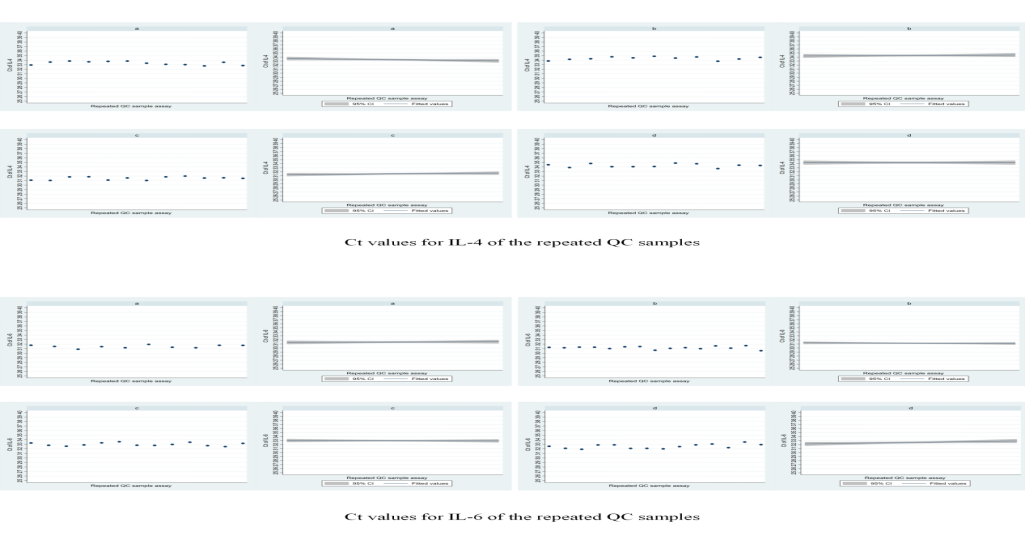  **H**  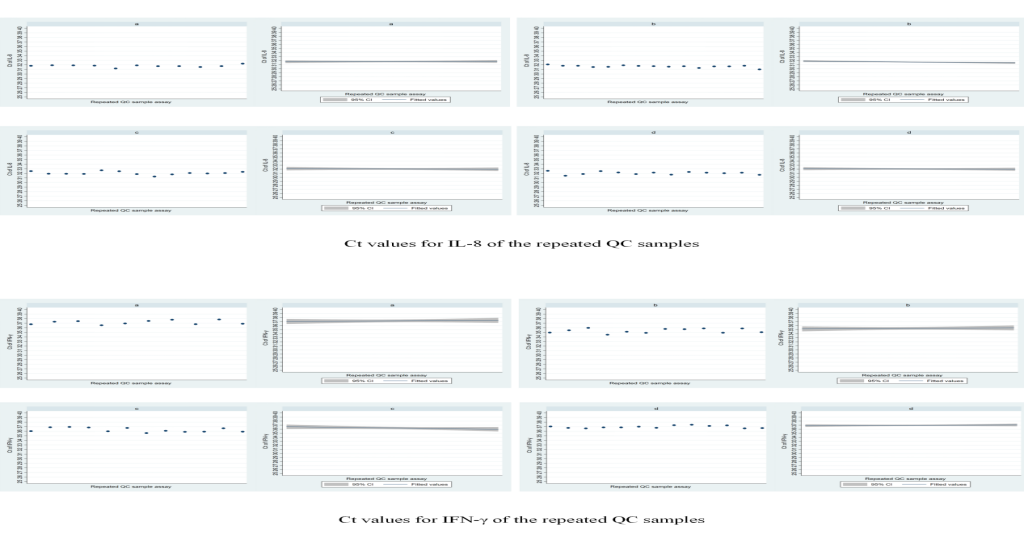 |
| **No Template Control (NTC) in each PCR plate** | 1. we validated the primers to ensure the stability of the amplification curves and and melting curves.  2. we set 1 NTC in each plate, considering that this study is a large sample multi-cytokine assay, thus we randomly chose one well of the target gene in the QC sample as the NTC each time (without QC sample template-replaced by sterile enzyme-free water), and the results of this group were considered reliable only if the NTC of the QC group showed a negative signal. |  |

**Table S3** Detailed information on confounders.

| **Detailed information on confounders.** |
| --- |
| Information on **maternal age,** **family monthly income per capita**, **alcohol and tobacco use during pregnancy** and **father’s education level** were reported by questionnaires. |
| Maternal alcohol consumption 1-2 times per month and more during pregnancy was defined as **maternal alcohol use during pregnancy**. |
| **Maternal smoking during pregnancy** was defined as a history of one or more smoking incidents while pregnant. |
| **Maternal pre-pregnancy BMI** was calculated by BMI (kg/m^2^) = weight (kg) / [height (m)]^2^ that measured by physicians at the first antenatal visit. |
| Data on **parity, maternal fever during pregnancy, maternal infection or inflammation conditions during pregnancy, metabolic dysfunctions during pregnancy, gestational week, birth weight, placental weight and children’s sex** were abstracted from the medical notes. |
| **Maternal metabolic dysfunctions** included hypertensive disorders during pregnancy and gestational diabetes. |
| **History of infection or inflammation conditions** in the mother during pregnancy covered tonsil/nose/throat swelling by colds or respiratory infections, vaginitis and pelvic inflammatory disease, as well as other inflammations like bronchitis, gastroenteritis, cholecystitis, and gastritis. |
| The Wechsler Adult Intelligence Scale-Revised by China (WAIS-RC) was adopted to measure the **mother’s IQ** during pregnancy. |
| **Placental efficiency** was calculated by the ratio of placental weight to birth weight. |
| Breastfeeding duration ≥ 6 months was defined as **exclusive breastfeeding**. |

**Table S4** Comparisons of the basic demographic characteristics of the included and excluded populations.

| **Variables** | **Included (n=1,665)** | **Excluded (n=1,608)** | **P-value** |
| --- | --- | --- | --- |
| **Maternal characteristics** |  |  |  |
| Age at enrollment (years) (Mean ± SD) | 26.4 ± 3.6 | 26.4 ± 3.7 | 0.760 |
| Parity [n (%)] |  |  | 0.519 |
| Nulliparous | 1507 (90.5) | 1444 (89.81) |  |
| Multipara | 158 (9.5) | 164 (10.2) |  |
| Pre-pregnancy BMI (kg/m^2^) (Mean ± SD) | 20.9 ± 2.9 | 20.9 ± 2.9 | 0.701 |
| Maternal metabolic dysfunctions during pregnancy [n (%)] |  |  | 0.645 |
| Yes | 285 (17.1) | 286 (17.8) |  |
| No | 1380 (82.9) | 1322 (82.2) |  |
| Maternal infection or inflammation conditions during pregnancy [n (%)] |  |  | 0.106 |
| Yes | 145 (8.7) | 115 (7.2) |  |
| No | 1520(91.3) | 1493 (92.8) |  |
| Maternal fever during pregnancy [n (%)] |  |  | 0.752 |
| Yes | 213(12.8) | 199 (12.4) |  |
| No | 1452(87.2) | 1409 (87.6) |  |
| Alcohol use during pregnancy [n (%)] |  |  | 0.402 |
| No | 1539 (92.4) | 1473 (91.6) |  |
| Yes | 126 (7.6) | 135 (8.4) |  |
| **Father’s characteristics** |  |  |  |
| Father’s educational levels [n (%)] |  |  | 0.032 |
| Junior high school or below | 223 (13.4) | 261 (16.2) |  |
| Senior middle school | 487 (29.2) | 424 (26.4) |  |
| Junior college or above | 955 (57.4) | 923 (57.4) |  |
| Family monthly income per capita, RMB/yuan [n (%)] |  |  | 0.843 |
| ＜2500 | 440 (26.4) | 420 (26.1) |  |
| ≥2500 | 1225 (73.6) | 1188 (73.9) |  |
| **Children’s characteristics** |  |  |  |
| Sex [n (%)] |  |  | 0.529 |
| Boys | 859 (51.6) | 797 (49.6) |  |
| Girls | 806 (48.4) | 811 (50.4) |  |
| Birth weight (g) (Mean ± SD) | 3384.4 ± 427.1 | 3344.3± 465.9 | 0.010 |
| Children’s age at cognition testing (months) (Mean ± SD) | 55.4 ± 6.8 | 56.1 ± 7.1 | 0.067 |

Abbreviations: IQ, intelligence quotient; SD, standard deviation.

**Table S5** Ct of internal reference genes and target genes in placental qPCR assays in the included population.

| **Ct of internal reference genes and target genes in the placenta** | **Ct** | |
| --- | --- | --- |
|  | **Mean** | **SD** |
| 18S | 11.88 | 2.38 |
| IL-8 | 31.72 | 1.99 |
| IL-1β | 32.77 | 2.45 |
| IL-6 | 32.04 | 2.42 |
| TNF-α | 33.22 | 2.43 |
| CRP | 34.69 | 3.13 |
| IFN-γ | 36.59 | 2.51 |
| IL-4 | 33.16 | 2.04 |
| IL-10 | 34.53 | 2.55 |

Abbreviations: Ct, cycle threshold; IL, interleukin; CRP, C-reactive protein; TNF-α, Tumor necrosis factor-alpha; IFN-γ, Interferon-gamma; SD, standard deviation.**Table S6** Comparison of cognitive scores in children with and without placental data.

| **Scores of WPPSI-Ⅳ** (mean; SD) | **Individuals with placental data available (n=1665)** | **Individuals without placental data available (n=497)** | **P-value** |
| --- | --- | --- | --- |
| VCI | 109.0(12.7) | 108.3(11.7) | 0.257 |
| VSI | 106.6(12.8) | 105.3(13.0) | 0.051 |
| FRI^#^ | 104.8(11.4) | 104.3(11.6) | 0.388 |
| WMI | 103.4(11.9) | 102.2(11.1) | 0.037 |
| PSI^#^ | 101.9(10.9) | 101.2(10.6) | 0.292 |
| FSIQ | 107.8(11.2) | 106.8(10.5) | 0.086 |

Abbreviations: IL, interleukin; CRP, C-reactive protein; TNF-α, Tumor necrosis factor-alpha; IFN-γ, Interferon-gamma; FSIQ, full scale intelligence quotient; VCI, verbal comprehension index; VSI, visual spatial index; FRI, fluid reasoning index; WMI, working memory index; PSI, processing speed index; ΔCt, Delta Ct.; SE, standard error.

^#^ There were 206 missing data in FRI and PSI in the inclusion population, and 60 missing data in FRI and PSI in individuals without placental data available.

**Table S7** The association between placental eight cytokines mRNA expression and children’s cognitive performance in a model by multiple linear regression model.

| **Cytokines** | **VCI** | **VSI** | **FRI** | **WMI** | **PSI** | **FSIQ** |
| --- | --- | --- | --- | --- | --- | --- |
|  | **Adjusted β (95%CI)**† | | | | | |
| IL-8 | -0.32(-0.93,0.29) | **-0.74(-1.37,-0.11)*** | **-0.75(-1.34,-0.15)*** | -0.03(-0.62,0.57) | -0.25(-0.83,0.33) | -0.43(-0.97,0.11) |
| IL-1β | 0.45(-0.06,0.96)^#^ | 0.40(-0.12,0.92) | 0.25(-0.25,0.75) | 0.09(-0.40,0.58) | -0.04(-0.52,0.45) | 0.35(-0.10,0.80) |
| IL-6 | -0.13(-0.72,0.46) | -0.14(-0.75,0.47) | -0.42(-1.01,0.17) | -0.39(-0.96,0.18) | **-0.58(-1.16,-0.01)*** | -0.44(-0.96,0.09) |
| TNF-α | -0.33(-0.86,0.21) | 0.33(-0.23,0.88) | 0.27(-0.26,0.80) | -0.06(-0.58,0.46) | 0.16(-0.35,0.67) | 0.07(-0.41,0.55) |
| CRP | -0.08(-0.38,0.22) | -0.06(-0.37,0.25) | 0.20(-0.10,0.49) | 0.16(-0.13,0.45) | -0.01(-0.30,0.28) | -0.02(-0.29,0.25) |
| IFN-γ | -0.02(-0.43,0.38) | -0.32(-0.73,0.10) | -0.11(-0.51,0.28) | **-0.42(-0.81,-0.03)*** | -0.33(-0.71,0.06)^#^ | -0.23(-0.59,0.13) |
| IL-4 | 0.11(-0.36,0.59) | 0.30(-0.19,0.79) | 0.36(-0.11,0.82) | 0.14(-0.49,0.39) | 0.28(-0.17,0.73) | 0.23(-0.19,0.66) |
| IL-10 | -0.04(-0.49,0.41) | -0.25(-0.72,0.22) | -0.40(-0.85,0.05) | -0.05(-0.49,0.39) | 0.32(-0.12,0.76) | -0.20(-0.60,0.21) |

Abbreviations: CI, confidence interval; IL, interleukin; CRP, C-reactive protein; TNF-α, Tumor necrosis factor-alpha; IFN-γ, Interferon-gamma; FSIQ, full scale intelligence quotient; VCI, verbal comprehension index; VSI, visual spatial index; FRI, fluid reasoning index; WMI, working memory index; PSI, processing speed index.

†adjusted for maternal age, maternal IQ, family monthly income per capita, pre-pregnancy BMI, parity, maternal metabolic dysfunctions, maternal fever during pregnancy, maternal infection or inflammation conditions during pregnancy, maternal alcohol use during pregnancy, father’s education level, children’s gender, and placental efficiency.

**^*^**: P < 0.05; **^**^**: P < 0.01.

**Table S8** The association between interaction of placental inflammatory cytokines mRNA expression*sex and children’s cognitive performance.

| **Cytokines** | **Models** |  | **VCI** | **VSI** | **FRI** | **WMI** | **PSI** | **FSIQ** |
| --- | --- | --- | --- | --- | --- | --- | --- | --- |
|  |  |  | **β (95%CI)** | | | | | |
| IL-8 | **Model 1** | IL-8*sex | -0.21(-0.49,0.06) | -0.31(-0.59,-0.03) | -0.34(-0.60,-0.07) | -0.09(-0.35,0.18) | -0.07(-0.33,0.19) | -0.25(-0.49,0.00) |
|  |  | P-value for interaction | **0.132** | **0.029** | **0.014** | 0.516 | 0.593 | **0.050** |
|  | **Model 2** | IL-8*sex | -0.18(-1.03,0.68) | **0.74(-0.15,1.62)** | 0.03(-0.81,0.87) | **0.73(-0.10,1.56)** | 0.49(-0.33,1.30) | 0.41(-0.35,1.17) |
|  |  | P-value for interaction | 0.687 | **0.104** | 0.321 | **0.084** | 0.242 | 0.293 |
| IL-1β | **Model 1** | IL-1β*sex | 0.16(-0.11,0.43) | -0.07(-0.34,0.21) | 0.01(-0.24,0.27) | 0.11(-0.14,0.36) | 0.11(-0.14,0.36) | 0.05(-0.18,0.29) |
|  |  | P-value for interaction | 0.236 | 0.628 | 0.915 | 0.400 | 0.383 | 0.687 |
|  | **Model 2** | IL-1β*sex | 0.12(-0.70,0.95) | -0.37(-1.22,0.49) | 0.04(-0.77,0.85) | 0.36(-0.44,1.16) | -0.13(-0.91,0.65) | 0.009-0.73,0.73) |
|  |  | P-value for interaction | 0.768 | 0.401 | 0.922 | 0.375 | 0.742 | 1.000 |
| IL-6 | **Model 1** | IL-6*sex | -0.30(-0.59,-0.02) | -0.16(-0.45,0.13) | -0.22(-0.49,0.06) | 0.15(-0.42,0.13) | -0.17(-0.43,0.09) | -0.31(-0.57,-0.06) |
|  |  | P-value for interaction | **0.039** | 0.287 | **0.121** | 0.295 | 0.202 | **0.017** |
|  | **Model 2** | IL-6*sex | -0.88(-1.76,0.00) | 0.70(-0.21,1.62) | 0.58(-0.28,1.45) | 0.72(-0.14,1.57) | -0.05(-0.89,0.79) | 0.02(-0.77,0.80) |
|  |  | P-value for interaction | **0.049** | **0.133** | 0.185 | **0.099** | 0.908 | 0.970 |
| TNF-α | **Model 1** | TNF-α*sex | -0.23(-0.48,0.01) | -0.13(-0.38,0.12) | -0.12(-0.36,0.11) | -0.08(-0.31,0.15) | 0.01(-0.22,0.23) | -0.17(-0.39,0.05) |
|  |  | P-value for interaction | **0.061** | 0.295 | 0.301 | 0.510 | 0.937 | **0.132** |
|  | **Model 2** | TNF-α*sex | -0.78(-1.55,-0.01) | -0.11(-0.91,0.70) | -0.60(-1.35,0.16) | 0.40(-0.36,1.15) | -0.13(-0.86,0.60) | -0.32(-1.01,0.37) |
|  |  | P-value for interaction | **0.048** | 0.796 | **0.120** | 0.302 | 0.726 | 0.357 |
| CRP | **Model 1** | CRP*sex | 0.02(-0.15,0.19) | -0.10(-0.27,0.07) | 0.11(-0.06,0.28) | 0.16(0.00,0.32) | 0.19(0.03,0.35) | 0.02(-0.13,0.17) |
|  |  | P-value for interaction | 0.785 | 0.257 | 0.189 | **0.047** | **0.019** | 0.775 |
|  | **Model 2** | CRP*sex | 0.39(-0.14,0.91) | -0.04(-0.58,0.51) | 0.28(-0.24,0.80) | 0.37(-0.14,0.88) | 0.74(0.24,1.25) | 0.37(-0.10,0.84) |
|  |  | P-value for interaction | **0.148** | 0.886 | 0.295 | 0.153 | **0.004** | **0.123** |
| IFN-γ | **Model 1** | IFN-γ*sex | -0.10(-0.32,0.12) | -0.26(-0.48,-0.04) | -0.13(-0.34,0.08) | -0.18(-0.38,0.03) | -0.06(-0.26,0.14) | -0.15(-0.34,0.04) |
|  |  | P-value for interaction | 0.372 | **0.019** | 0.216 | **0.087** | 0.544 | **0.124** |
|  | **Model 2** | IFN-γ*sex | -0.23(-0.89,0.43) | -0.19(-0.88,0.49) | -0.28(-0.93,0.37) | 0.27(-0.37,0.90) | 0.32(-0.32,0.95) | 0.11(-0.48,0.70) |
|  |  | P-value for interaction | 0.497 | 0.580 | 0.402 | 0.415 | 0.328 | 0.709 |
| IL-4 | **Model 1** | IL-4*sex | -0.11(-0.36,0.14) | -0.16(-0.41,0.09) | -0.08(-0.32,0.17) | -0.04(-0.27,0.20) | 0.06(-0.18,0.29) | -0.09(-0.31,0.14) |
|  |  | P-value for interaction | 0.406 | 0.215 | 0.527 | 0.753 | 0.622 | 0.445 |
|  | **Model 2** | IL-4*sex | -0.22(-0.98,0.55) | 0.00(-0.79,0.79) | -0.50(-1.26,0.26) | 0.26(-0.48,1.00) | -0.05(-0.79,0.69) | 0.09(-0.59,0.77) |
|  |  | P-value for interaction | 0.582 | 0.998 | 0.195 | 0.495 | 0.892 | 0.796 |
| IL-10 | **Model 1** | IL-10*sex | 0.04(-0.20,0.29) | -0.15(-0.40,0.10) | -0.04(-0.28,0.20) | 0.13(-0.10,0.36) | 0.28(0.05,0.51) | -0.03(-0.24,0.19) |
|  |  | P-value for interaction | 0.725 | 0.230 | 0.726 | 0.264 | **0.016** | 0.823 |
|  | **Model 2** | IL-10*sex | -0.01(-0.76,0.74) | -0.01(-0.78,0.77) | 0.55(-0.20,1.29) | 0.58(-0.15,1.30) | 0.46(-0.26,1.18) | 0.32(-0.34,0.99) |
|  |  | P-value for interaction | 0.986 | 0.998 | **0.150** | **0.119** | 0.213 | 0.342 |

Abbreviations: CI, confidence interval; IL, interleukin; CRP, C-reactive protein; TNF-α, Tumor necrosis factor-alpha; IFN-γ, Interferon-gamma; FSIQ, full scale intelligence quotient; VCI, verbal comprehension index; VSI, visual spatial index; FRI, fluid reasoning index; WMI, working memory index; PSI, processing speed index.

Model 1: crude models.

Model 2: adjusted for maternal age, maternal IQ, family monthly income per capita, pre-pregnancy BMI, parity, maternal metabolic dysfunctions, maternal fever during pregnancy, maternal infection or inflammation conditions during pregnancy, maternal alcohol use during pregnancy, father’s education level, children’s gender, and placental efficiency.

Note: P-value for interaction < 0.15 was considered to have sex interaction with the independent variables.

**Table S9** Sensitivity analysis of the association between placental summary index of cytokines and children’s cognitive performance by multivariate linear regression analyses.

| **Summary index of cytokines** | **Participants** | **Sensitivity analysis** | **VCI** | **VSI** | **FRI** | **WMI** | **PSI** | **FSIQ** |
| --- | --- | --- | --- | --- | --- | --- | --- | --- |
|  |  |  | **Adjusted β (95%CI)** | | | | | |
| **Index 1** | **Total ^a^** | **Sensitivity Analysis 1** | -0.18(-0.66,0.31) | -0.34(-0.85,0.16) | -0.38(-0.87,0.12) | -0.20(-0.68,0.27) | 0.07(-0.41,0.55) | -0.35(-0.78,0.09) |
|  |  | P-value | 0.483 | 0.183 | 0.135 | 0.398 | 0.773 | 0.118 |
|  | **Boys ^b^** | **Sensitivity Analysis 1** | -0.14(-0.88,0.59) | -0.42(-1.17,0.34) | -0.42(-1.12,0.29) | -0.61(-1.30,0.08) | -0.09(-0.77,0.60) | -0.59(1.25,0.07) |
|  |  | P-value | 0.701 | 0.277 | 0.251 | 0.085 | 0.804 | 0.078 |
|  | **Girls ^b^** | **Sensitivity Analysis 1** | -0.23(-0.88,0.41) | -0.29(-0.97,0.40) | -0.27(-0.95,0.41) | 0.21(-0.43,0.86) | 0.26(-0.42,0.94) | -0.12(-0.68,0.45) |
|  |  | P-value | 0.478 | 0.409 | 0.429 | 0.518 | 0.450 | 0.690 |
|  | **Total ^c^** | **Sensitivity Analysis 2** | -0.16(-0.66,0.34) | -0.33(-0.85,0.19) | -0.32(-0.82,0.18) | -0.22(-0.70,0.27) | 0.09(-0.39,0.58) | -0.34(-0.78,0.11) |
|  |  | P-value | 0.537 | 0.209 | 0.206 | 0.377 | 0.704 | 0.138 |
|  | **Boys ^d^** | **Sensitivity Analysis 2** | -0.06(-0.82,0.69) | -0.38(-1.14,0.39) | -0.33(-1.05,0.39) | -0.59(-1.30,0.12) | -0.05(-0.74,0.64) | -0.53(-1.21,0.14) |
|  |  | P-value | 0.868 | 0.335 | 0.364 | 0.106 | 0.884 | 0.121 |
|  | **Girls ^d^** | **Sensitivity Analysis 2** | -0.25(-0.91,0.40) | -0.31(-1.00,0.39) | -0.26(-0.95,0.43) | 0.16(-0.50,0.82) | 0.27(-0.41,0.95) | -0.14(-0.72,0.44) |
|  |  | P-value | 0.445 | 0.387 | 0.457 | 0.639 | 0.438 | 0.636 |
| **Index 2** | **Total ^a^** | **Sensitivity Analysis 1** | **-0.60(-1.16,-0.05)** | **-0.66(-1.24,-0.08)** | **-0.60(-1.15,-0.04)** | **-0.65(-1.19,-0.11)** | **-0.55(-1.09,-0.01)** | **-0.78(-1.27,-0.28)** |
|  |  | P-value | 0.034 | 0.027 | 0.036 | 0.018 | 0.048 | 0.002 |
|  | **Boys ^b^** | **Sensitivity Analysis 1** | -0.52(-1.36,0.32) | -0.84(-1.70,0.01) | -0.63(-1.44,0.18) | **-1.26(-2.05,-0.48)** | **-0.93(-1.71,-0.16)** | **-1.05(-1.80,-0.30)** |
|  |  | P-value | 0.224 | 0.053 | 0.125 | 0.002 | 0.018 | 0.006 |
|  | **Girls ^b^** | **Sensitivity Analysis 1** | -0.72(-1.46,0.03) | -0.52(-1.31,0.27) | -0.53(-1.29,0.24) | -0.04(-0.79,0.71) | -0.12(-0.88,0.64) | -0.53(-1.19,0.12) |
|  |  | P-value | 0.059 | 0.199 | 0.175 | 0.915 | 0.759 | 0.111 |
|  | **Total ^c^** | **Sensitivity Analysis 2** | -0.45(-1.02,0.12) | -0.52(-1.10,0.08) | -0.48(-1.04,0.09) | -0.53(-1.08,0.02) | -0.48(-1.03,0.06) | **-0.65(-1.16,-0.15)** |
|  |  | P-value | 0.121 | 0.087 | 0.096 | 0.059 | 0.081 | 0.012 |
|  | **Boys ^d^** | **Sensitivity Analysis 2** | -0.29(-1.14,0.57) | -0.59(-1.46,0.27) | -0.48(-1.29,0.34) | **-1.06(-1.86,-0.26)** | **-0.87(-1.65,-0.09)** | **-0.84(-1.61,-0.08)** |
|  |  | P-value | 0.513 | 0.179 | 0.252 | 0.009 | 0.029 | 0.030 |
|  | **Girls ^d^** | **Sensitivity Analysis 2** | -0.64(-1.40,0.11) | -0.43(-1.23,0.37) | -0.46(-1.23,0.32) | 0.01(-0.76,0.77) | -0.07(-0.84,0.70) | -0.47(-1.14,0.19) |
|  |  | P-value | 0.096 | 0.294 | 0.245 | 0.985 | 0.853 | 0.164 |

Abbreviations: CI, confidence interval; Interferon-gamma; FSIQ, full scale intelligence quotient; VCI, verbal comprehension index; VSI, visual spatial index; FRI, fluid reasoning index; WMI, working memory index; PSI, processing speed index.

Index 1: the summary index of IL-10 and IL-4; Index 2: the summary index of IL-8, IL-6, TNF-α, IL-1β, CRP, and IFN-γ.

**Sensitivity Analysis 1:**

**^a^**adjusted for maternal age, maternal IQ, family monthly income per capita, pre-pregnancy BMI, parity, maternal metabolic dysfunctions, maternal fever during pregnancy, maternal infection or inflammation conditions during pregnancy, maternal alcohol use during pregnancy, father’s education level, children’s gender, placental efficiency. + Children’s age at cognition assessment.

**^b^**adjusted for maternal age, maternal IQ, family monthly income per capita, pre-pregnancy BMI, parity, maternal metabolic dysfunctions, maternal fever during pregnancy, maternal infection or inflammation conditions during pregnancy, maternal alcohol use during pregnancy, father’s education level, placental efficiency. + Children’s age at cognition assessment.

**Sensitivity Analysis 2:**

**^c^**adjusted for maternal age, maternal IQ, family monthly income per capita, pre-pregnancy BMI, parity, maternal metabolic dysfunctions, maternal fever during pregnancy, maternal infection or inflammation conditions during pregnancy, maternal alcohol use during pregnancy, father’s education level, children’s gender, placental efficiency. + exclusive breastfeeding for the first six months+main caregivers before 3 years+average screen time per day+average outdoor activity time per day.

**^c^**adjusted for maternal age, maternal IQ, family monthly income per capita, pre-pregnancy BMI, parity, maternal metabolic dysfunctions, maternal fever during pregnancy, maternal infection or inflammation conditions during pregnancy, maternal alcohol use during pregnancy, father’s education level, placental efficiency. + exclusive breastfeeding for the first six months+main caregivers before 3 years+average screen time per day+average outdoor activity time per day.

^*^: P < 0.05; ^**^: P < 0.01.

**Table S10** Sensitivity analysis of the association between placental each inflammatory cytokine mRNA expression and children’s cognitive performance by multivariate linear regression models.

| **Cytokines** | | **VCI** | **VSI** | **FRI** | **WMI** | **PSI** | **FSIQ** |
| --- | --- | --- | --- | --- | --- | --- | --- |
|  |  | **Adjusted β (95%CI)** | | | | | |
| IL-8 | **Sensitivity Analysis 1** | -0.35(-0.77,0.07) | **-0.53(-0.96,-0.09)** | **-0.61(-1.03,0.19)^★^** | -0.31(-0.72,0.09) | -0.36(-0.77,0.04) | **-0.50(-0.87,-0.12)^★^** |
|  | P-value | 0.105 | 0.018 | 0.004 | 0.132 | 0.079 | 0.009 |
|  | **Sensitivity Analysis 2** | -0.39(-0.82,0.04) | **-0.57(-1.01,-0.12)** | **-0.62(-1.04,-0.20)^★^** | -0.37(-0.79,0.05) | -0.38(-0.79,0.03) | **-0.54(-0.92,-0.16)^★^** |
|  | P-value | 0.074 | 0.013 | 0.004 | 0.081 | 0.069 | 0.006 |
| IL-1β | **Sensitivity Analysis 1** | 0.08(-0.33,0.48) | -0.07(-0.49,0.36) | -0.12(-0.52,0.28) | -0.11(-0.50,0.29) | -0.09(-0.48,0.30) | -0.08(-0.44,0.29) |
|  | P-value | 0.713 | 0.760 | 0.543 | 0.594 | 0.658 | 0.683 |
|  | **Sensitivity Analysis 2** | 0.15(-0.26,0.57) | 0.01(-0.42,0.44) | -0.08(-0.48,0.33) | -0.03(-0.43,0.37) | -0.06(-0.45,0.33) | -0.01(-0.38,0.36) |
|  | P-value | 0.465 | 0.970 | 0.715 | 0.899 | 0.769 | 0.949 |
| IL-6 | **Sensitivity Analysis 1** | **-0.60(-1.04,-0.17)^★^** | **-0.45(-0.90,0.00)** | **-0.64(-1.07,-0.21)^★^** | **-0.66(-1.08,-0.24)^★^** | **-0.52(-0.94,-0.11)** | **-0.73(-1.12,-0.35)^★^** |
|  | P-value | 0.007 | 0.052 | 0.003 | 0.002 | 0.014 | 0.001 |
|  | **Sensitivity Analysis 2** | -0.40(-0.84,0.04) | -0.27(-0.73,0.19) | **-0.53(-0.96,-0.10)** | **-0.51(-0.94,-0.08)** | **-0.47(-0.89,-0.05)** | **-0.58(-0.97,-0.18)^★^** |
|  | P-value | 0.078 | 0.257 | 0.017 | 0.020 | 0.030 | 0.004 |
| TNF-α | **Sensitivity Analysis 1** | **-0.49(-0.87,-0.11)^★^** | -0.17(-0.58,0.23) | -0.29(-0.66,0.09) | **-0.44(-0.80,-0.07)^★^** | -0.27(-0.64,0.09) | **-0.41(-0.75,-0.07)^★^** |
|  | P-value | 0.012 | 0.397 | 0.133 | 0.016 | 0.142 | 0.018 |
|  | **Sensitivity Analysis 2** | **-0.42(-0.81,-0.03)** | -0.11(-0.52,0.29) | -0.25(-0.62,0.13) | **-0.40(-0.77,-0.02)** | -0.25(-0.62,0.12) | **-0.36(-0.71,-0.02)** |
|  | P-value | 0.033 | 0.579 | 0.202 | 0.038 | 0.179 | 0.040 |
| CRP | **Sensitivity Analysis 1** | -0.15(-0.41,0.11) | -0.17(-0.44,0.10) | 0.01(-0.25,0.27) | 0.02(-0.23,0.27) | -0.01(-0.27,0.24) | -0.14(-0.37,0.09) |
|  | P-value | 0.259 | 0.211 | 0.914 | 0.886 | 0.913 | 0.228 |
|  | **Sensitivity Analysis 2** | -0.05(-0.31,0.22) | -0.08(-0.35,0.20) | 0.09(-0.17,0.35) | 0.11(-0.14,0.37) | 0.03(-0.22,0.29) | -0.06(-0.30,0.17) |
|  | P-value | 0.727 | 0.576 | 0.506 | 0.392 | 0.800 | 0.612 |
| IFN-γ | **Sensitivity Analysis 1** | -0.14(-0.46,0.18) | -0.32(-0.66,0.01) | -0.21(-0.53,0.12) | **-0.41(-0.72,-0.10)^★^** | **-0.31(-0.62,0.01)** | **-0.29(-0.58,-0.01)** |
|  | P-value | 0.396 | 0.059 | 0.206 | 0.010 | 0.054 | 0.047 |
|  | **Sensitivity Analysis 2** | -0.17(-0.50,0.16) | **-0.36(-0.70,-0.02)** | -0.20(-0.53,0.13) | **-0.44(-0.76,-0.12)^★^** | **-0.32(-0.63,0.00)** | **-0.31(-0.61,-0.02)** |
|  | P-value | 0.300 | 0.041 | 0.226 | 0.005 | 0.049 | 0.038 |
| IL-4 | **Sensitivity Analysis 1** | -0.07(-0.45,0.30) | -0.08(-0.47,0.31) | -0.05(-0.42,0.33) | -0.10(-0.47,0.26) | -0.04(-0.41,0.32) | -0.11(-0.45,0.23) |
|  | P-value | 0.704 | 0.676 | 0.814 | 0.582 | 0.820 | 0.521 |
|  | **Sensitivity Analysis 2** | -0.15(-0.53,0.24) | -0.15(-0.55,0.24) | -0.05(-0.43,0.33) | -0.20(-0.57,0.17) | -0.06(-0.43,0.31) | -0.17(-0.51,0.17) |
|  | P-value | 0.449 | 0.451 | 0.796 | 0.296 | 0.763 | 0.324 |
| IL-10 | **Sensitivity Analysis 1** | -0.13(-0.50,0.24) | -0.32(-0.70,0.07) | **-0.38(-0.75,-0.01)** | -0.14(-0.49,0.22) | 0.12(-0.24,0.48) | -0.29(-0.62,0.04) |
|  | P-value | 0.490 | 0.108 | 0.043 | 0.460 | 0.509 | 0.082 |
|  | **Sensitivity Analysis 2** | -0.04(-0.41,0.34) | -0.23(-0.62,0.16) | -0.31(-0.69,0.06) | -0.06(-0.43,0.31) | 0.16(-0.20,0.52) | -0.22(-0.55,0.12) |
|  | P-value | 0.851 | 0.247 | 0.099 | 0.757 | 0.385 | 0.203 |

Abbreviations: CI, confidence interval; IL, interleukin; CRP, C-reactive protein; TNF-α, Tumor necrosis factor-alpha; IFN-γ, Interferon-gamma; FSIQ, full scale intelligence quotient; VCI, verbal comprehension index; VSI, visual spatial index; FRI, fluid reasoning index; WMI, working memory index; PSI, processing speed index.

Sensitivity Analysis 1: adjusted for maternal age, maternal IQ, family monthly income per capita, pre-pregnancy BMI, parity, maternal metabolic dysfunctions, maternal fever during pregnancy, maternal infection or inflammation conditions during pregnancy, maternal alcohol use during pregnancy, father’s education level, children’s gender, placental efficiency. + Children’s age at cognition assessment.

Sensitivity Analysis 2: adjusted for maternal age, maternal IQ, family monthly income per capita, pre-pregnancy BMI, parity, maternal metabolic dysfunctions, maternal fever during pregnancy, maternal infection or inflammation conditions during pregnancy, maternal alcohol use during pregnancy, father’s education level, children’s gender, placental efficiency. + exclusive breastfeeding for the first six months+main caregivers before 3 years+average screen time per day+average outdoor activity time per day.

**^★^**: P-_FDR_ < 0.05.

**Table S11** Sensitivity analysis of the association between placental cytokine mRNA expression and children’s cognitive performance by sex.

| **Cytokines** | **Sensitivity analyses** | **Sex** | VCI | VSI | FRI | WMI | PSI | FSIQ |
| --- | --- | --- | --- | --- | --- | --- | --- | --- |
|  |  |  | **Adjusted β (95%CI)** | | | | | |
| IL-8 |  |  |  |  |  |  |  |  |
|  | **Sensitivity analysis 1** | Boys | -0.26(-0.90,0.38) | **-0.94(-1.60,-0.29)^★^** | **-0.63(-1.24,-0.02)** | **-0.72(-1.32,-0.12)^★^** | **-0.63(-1.22,-0.04)** | **-0.74(-1.31,-0.17)^★^** |
|  | P-value |  | 0.426 | 0.005 | 0.044 | 0.019 | 0.038 | 0.012 |
|  | **Sensitivity analysis 2** | Boys | -0.32(-0.98,0.34) | **-0.97(-1.63,-0.30)^★^** | **-0.63(-1.25,-0.01)** | **-0.77(-1.38,-0.15)^★^** | **-0.64(-1.24,-0.05)** | **-0.77(-1.36,-0.19)** |
|  | P-value |  | 0.345 | 0.004 | 0.047 | 0.015 | 0.034 | 0.010 |
|  | **Sensitivity analysis 1** | Girls | -0.43(-0.97,0.12) | -0.20(-0.78,0.38) | **-0.63(-1.20,-0.07)** | -0.02(-0.53,0.58) | -0.14(-0.70,0.43) | -0.32(-0.80,0.17) |
|  | P-value |  | 0.128 | 0.503 | 0.028 | 0.932 | 0.635 | 0.199 |
|  | **Sensitivity analysis 2** | Girls | -0.48(-1.04,0.08) | -0.23(-0.82,0.36) | **-0.66(-1.23,-0.09)** | -0.06(-0.62,0.51) | -0.16(-0.72,0.41) | -0.36(-0.85,0.13) |
|  | P-value |  | 0.092 | 0.450 | 0.023 | 0.847 | 0.593 | 0.150 |
| IL-1β |  |  |  |  |  |  |  |  |
|  | **Sensitivity analysis 1** | Boys | 0.00(-0.60,0.61) | 0.06(-0.56,0.67) | -0.24(-0.81,0.33) | -0.33(-0.89,0.24) | -0.03(-0.58,0.52) | -0.12(-0.66,0.42) |
|  | P-value |  | 0.989 | 0.851 | 0.402 | 0.257 | 0.921 | 0.655 |
|  | **Sensitivity analysis 2** | Boys | 0.09(-0.52,0.71) | 0.16(-0.47,0.78) | -0.16(-0.74,0.41) | -0.25(-0.82,0.33) | 0.01(-0.54,0.56) | -0.05(-0.60,0.50) |
|  | P-value |  | 0.773 | 0.621 | 0.573 | 0.401 | 0.971 | 0.858 |
|  | **Sensitivity analysis 1** | Girls | 0.15(-0.39,0.69) | -0.21(-0.79,0.37) | -0.01(-0.58,0.55) | 0.97(-0.47,0.62) | -0.15(-0.71,0.42) | -0.05(-0.53,0.43) |
|  | P-value |  | 0.588 | 0.474 | 0.963 | 0.793 | 0.613 | 0.834 |
|  | **Sensitivity analysis 2** | Girls | 0.21(-0.34,0.76) | -0.16(-0.74,0.43) | 0.00(-0.57,0.57) | 0.15(-0.41,0.71) | -0.14(-0.70,0.43) | 0.00(-0.49,0.48) |
|  | P-value |  | 0.460 | 0.600 | 0.996 | 0.595 | 0.634 | 0.990 |
| IL-6 |  |  |  |  |  |  |  |  |
|  | **Sensitivity analysis 1** | Boys | -0.19(-0.85,0.47) | **-0.87(-1.54,-0.20)^★^** | **-0.91(-1.53,-0.28)^★^** | **-1.01(-1.63,-0.40)^★^** | -0.51(-1.12,0.09) | **-0.78(-1.37,-0.19)^★^** |
|  | P-value |  | 0.571 | 0.011 | 0.005 | 0.001 | 0.098 | 0.009 |
|  | **Sensitivity analysis 2** | Boys | 0.02(-0.65,0.69) | -0.65(-1.33,0.03) | **-0.80(-1.43,-0.16)** | **-0.84(-1.47,-0.21)^★^** | -0.46(-1.07,0.15) | **-0.60(-1.20,0.00)** |
|  | P-value |  | 0.947 | 0.062 | 0.014 | 0.009 | 0.135 | 0.049 |
|  | **Sensitivity analysis 1** | Girls | **-1.01(-1.58,-0.44)^★^** | -0.09(-0.70,0.52) | -0.38(-0.97,0.20) | -0.28(-0.86,0.30) | -0.53(-1.11,0.06) | **-0.69(-1.20,-0.19)^★^** |
|  | P-value |  | 0.001 | 0.783 | 0.200 | 0.340 | 0.077 | 0.003 |
|  | **Sensitivity analysis 2** | Girls | **-0.82(-1.41,-0.24)^★^** | 0.10(-0.52,0.72) | -0.30(-0.89,0.30) | -0.16(-0.75,0.44) | -0.47(-1.06,0.13) | -0.55(-1.06,-0.04) |
|  | P-value |  | 0.006 | 0.746 | 0.332 | 0.607 | 0.122 | 0.036 |
| TNF-α |  |  |  |  |  |  |  |  |
|  | **Sensitivity analysis 1** | Boys | -0.15(-0.73,0.42) | -0.18(-0.77,0.41) | 0.03(-0.51,0.58) | **-0.66(-1.20,-0.12)^★^** | -0.21(-0.74,0.31) | -0.29(-0.80,0.23) |
|  | P-value |  | 0.601 | 0.553 | 0.905 | 0.017 | 0.427 | 0.275 |
|  | **Sensitivity analysis 2** | Boys | -0.04(-0.63,0.55) | -0.06(-0.66,0.54) | 0.09(-0.46,0.64) | **-0.57(-1.12,-0.02)** | -0.19(-0.71,0.34) | -0.20(-0.72,0.33) |
|  | P-value |  | 0.900 | 0.851 | 0.746 | 0.044 | 0.491 | 0.467 |
|  | **Sensitivity analysis 1** | Girls | **-0.83(-1.33,-0.34)^★^** | -0.22(-0.75,0.31) | **-0.58(-1.09,-0.06)** | -0.19(-0.69,0.32) | -0.32(-0.83,0.20) | **-0.55(-0.99,-0.10)** |
|  | P-value |  | 0.001 | 0.415 | 0.028 | 0.462 | 0.227 | 0.016 |
|  | **Sensitivity analysis 2** | Girls | **-0.83(-1.33,-0.32)^★^** | -0.21(-0.75,0.33) | **-0.56(-1.08,-0.05)** | -0.20(-0.72,0.31) | -0.31(-0.83,0.21) | **-0.54(-0.97,-0.09)** |
|  | P-value |  | 0.001 | 0.447 | 0.033 | 0.438 | 0.238 | 0.019 |
| CRP |  |  |  |  |  |  |  |  |
|  | **Sensitivity analysis 1** | Boys | -0.38(-0.76,0.00) | -0.19(-0.58,0.20) | -0.10(-0.47,0.26) | -0.17(-0.53,0.19) | **-0.36(-0.71,-0.07)** | **-0.36(-0.70,-0.02)** |
|  | P-value |  | 0.052 | 0.335 | 0.583 | 0.343 | 0.046 | 0.041 |
|  | **Sensitivity analysis 2** | Boys | -0.23(-0.62,0.16) | -0.06(-0.45,0.34) | -0.03(-0.40,0.33) | -0.05(-0.42,0.32) | -0.32(-0.67,0.04) | -0.24(-0.59,0.11) |
|  | P-value |  | 0.239 | 0.771 | 0.859 | 0.788 | 0.079 | 0.179 |
|  | **Sensitivity analysis 1** | Girls | 0.09(-0.27,0.44) | -0.13(-0.50,0.25) | 0.20(-0.17,0.57) | 0.25(-0.11,0.60) | **0.42(0.05,0.78)** | 0.10(-0.21,0.41) |
|  | P-value |  | 0.635 | 0.511 | 0.295 | 0.171 | 0.025 | 0.512 |
|  | **Sensitivity analysis 2** | Girls | 0.17(-0.19,0.53) | -0.05(-0.43,0.33) | 0.27(-0.10,0.64) | 0.32(-0.04,0.68) | **0.47(0.11,0.84)** | 0.17(-0.15,0.48) |
|  | P-value |  | 0.349 | 0.803 | 0.154 | 0.084 | 0.012 | 0.299 |
| IFN-γ |  |  |  |  |  |  |  |  |
|  | **Sensitivity analysis 1** | Boys | -0.08(-0.55,0.40) | -0.28(-0.76,0.21) | -0.09(-0.56,0.37) | **-0.60(-1.05,-0.15)^★^** | **-0.46(-0.91,-0.02)** | -0.39(-0.82,0.04) |
|  | P-value |  | 0.757 | 0.262 | 0.698 | 0.009 | 0.042 | 0.072 |
|  | **Sensitivity analysis 2** | Boys | -0.06(-0.55,0.43) | -0.25(-0.75,0.24) | -0.05(-0.52,0.41) | **-0.59(-1.04,-0.13)^★^** | **-0.46(-0.91,-0.01)** | -0.36(-0.80,0.07) |
|  | P-value |  | 0.816 | 0.315 | 0.820 | 0.012 | 0.043 | 0.103 |
|  | **Sensitivity analysis 1** | Girls | -0.23(-0.66,0.21) | -0.40(-0.86,0.07) | -0.32(-0.77,0.12) | -0.22(-0.66,0.22) | -0.14(-0.59,0.31) | -0.22(-0.60,0.17) |
|  | P-value |  | 0.312 | 0.094 | 0.162 | 0.322 | 0.531 | 0.271 |
|  | **Sensitivity analysis 2** | Girls | -0.32(-0.76,0.12) | -0.47(-0.93,0.00) | -0.34(-0.79,0.12) | -0.30(-0.74,0.15) | -0.17(-0.62,0.29) | -0.28(-0.67,0.11) |
|  | P-value |  | 0.158 | 0.052 | 0.148 | 0.194 | 0.471 | 0.162 |
| IL-4 |  |  |  |  |  |  |  |  |
|  | **Sensitivity analysis 1** | Boys | -0.03(-0.54,0.59) | -0.09(-0.67,0.48) | 0.20(-0.34,0.74) | -0.26(-0.79,0.28) | 0.00(-0.52,0.52) | -0.17(-0.68,0.34) |
|  | P-value |  | 0.932 | 0.750 | 0.471 | 0.342 | 0.991 | 0.508 |
|  | **Sensitivity analysis 2** | Boys | -0.05(-0.63,0.53) | -0.17(-0.76,0.42) | 0.23(-0.32,0.78) | -0.35(-0.89.0.20) | -0.01(-0.53,0.52) | -0.23(-0.75,0.29) |
|  | P-value |  | 0.870 | 0.574 | 0.414 | 0.215 | 0.977 | 0.391 |
|  | **Sensitivity analysis 1** | Girls | -0.18(-0.67,0.32) | -0.08(-0.61,0.45) | -0.26(-0.79,0.26) | 0.06(-0.44,0.56) | -0.04(-0.56,0.48) | -0.05(-0.49,0.39) |
|  | P-value |  | 0.487 | 0.774 | 0.324 | 0.810 | 0.879 | 0.822 |
|  | **Sensitivity analysis 2** | Girls | -0.26(-0.77,0.24) | -0.14(-0.67,0.39) | -0.30(-0.83,0.23) | -0.05(-0.56,0.46) | -0.06(-0.59,0.46) | -0.12(-0.56,0.33) |
|  | P-value |  | 0.306 | 0.608 | 0.273 | 0.858 | 0.810 | 0.600 |
| IL-10 |  |  |  |  |  |  |  |  |
|  | **Sensitivity analysis 1** | Boys | -0.18(-0.73,0.37) | -0.37(-0.93,0.19) | **-0.64(-1.16,-0.12)** | -0.43(-0.95,0.08) | -0.09(-0.59,0.41) | **-0.50(-0.99,-0.01)** |
|  | P-value |  | 0.513 | 0.190 | 0.017 | 0.100 | 0.723 | 0.048 |
|  | **Sensitivity analysis 2** | Boys | -0.03(-0.59,0.54) | -0.26(-0.83,0.31) | **-0.57(-1.09,-0.04)** | -0.32(-0.85,0.20) | -0.05(-0.55,0.46) | -0.38(-0.88,0.13) |
|  | P-value |  | 0.929 | 0.375 | 0.034 | 0.229 | 0.852 | 0.142 |
|  | **Sensitivity analysis 1** | Girls | -0.10(-0.60,0.40) | -0.27(-0.79,0.26) | -0.06(-0.59,0.46) | 0.19(-0.31,0.69) | 0.35(-0.17,0.88) | -0.09(-0.52,0.35) |
|  | P-value |  | 0.687 | 0.322 | 0.816 | 0.448 | 0.186 | 0.695 |
|  | **Sensitivity analysis 2** | Girls | -0.04(-0.55,0.47) | -0.22(-0.76,0.31) | -0.01(-0.55,,0.52) | 0.24(-0.28,0.74) | 0.39(-0.14,0.92) | -0.05(-0.49,0.40) |
|  | P-value |  | 0.876 | 0.411 | 0.960 | 0.366 | 0.149 | 0.836 |

Abbreviations: CI, confidence interval; IL, interleukin; CRP, C-reactive protein; TNF-α, Tumor necrosis factor-alpha; IFN-γ, Interferon-gamma; FSIQ, full scale intelligence quotient; VCI, verbal comprehension index; VSI, visual spatial index; FRI, fluid reasoning index; WMI, working memory index; PSI, processing speed index.

**Sensitivity Analysis 1**: adjusted for maternal age, maternal IQ, family monthly income per capita, pre-pregnancy BMI, parity, maternal metabolic dysfunctions, maternal fever during pregnancy, maternal infection or inflammation conditions during pregnancy, maternal alcohol use during pregnancy, father’s education level, placental efficiency. + Children’s age at cognition assessment.

**Sensitivity Analysis 2**: adjusted for maternal age, maternal IQ, family monthly income per capita, pre-pregnancy BMI, parity, maternal metabolic dysfunctions, maternal fever during pregnancy, maternal infection or inflammation conditions during pregnancy, maternal alcohol use during pregnancy, father’s education level, placental efficiency,. + exclusive breastfeeding for the first six months+main caregivers before 3 years+average screen time per day+average outdoor activity time per day.

**^★^**: P-_FDR_ < 0.05.

**Table S12** Comparison of Ct of endogenous reference RNA-18S between sexes.

| **Sex** | **Ct** | | **P-value** |
| --- | --- | --- | --- |
|  | **Mean** | **SD** |  |
| Total test samples |  |  | 0.502 |
| Boys | 11.73 | 2.35 |  |
| Girls | 11.79 | 2.43 |  |
| Samples included in this study |  |  | 0.941 |
| Boys | 11.69 | 2.34 |  |
| Girls | 11.70 | 2.42 |  |
