# Supplementary material for: Placental inflammatory cytokines mRNA expression and preschool children’s cognitive performance: a birth cohort study in China
Source: BMC Med. 2023 Nov 20;21:449. doi: 10.1186/s12916-023-03173-2 (PMC10658981; doi:10.1186/s12916-023-03173-2)
Supplement: Supplementary file 2 — Additional file 2. STROBE checklist. [file 12916_2023_3173_MOESM2_ESM.docx]

STROBE Statement—Checklist of items that should be included in reports of ***cohort studies***

|  | | Item No | | Recommendation | Section (notes) | |  |
| --- | --- | --- | --- | --- | --- | --- | --- |
| **Title and abstract** | | 1 | | (*a*) Indicate the study’s design with a commonly used term in the title or the abstract | Title (“birth cohort study”) | |  |
|  |  |  |  | (*b*) Provide in the abstract an informative and balanced summary of what was done and what was found | Abstract: Methods and Results sections | |  |
| Introduction | | | | | | |  |
| Background/rationale | | 2 | | Explain the scientific background and rationale for the investigation being reported | Background section (paragraph 1 to  4) | |  |
| Objectives | | 3 | | State specific objectives, including any prespecified hypotheses | Background section (final paragraph) | |  |
| Methods | | | | | | |  |
| Study design | | 4 | | Present key elements of study design early in the paper | Methods section (paragraph 1) | |  |
| Setting | | 5 | | Describe the setting, locations, and relevant dates, including periods of recruitment, exposure, follow-up, and data collection | Methods section(“Study participants”section) | |  |
| Participants | | 6 | | (*a*) Give the eligibility criteria, and the sources and methods of selection of participants. Describe methods of follow-up | Methods (“Study participants”  sections) | |  |
|  |  |  |  | (*b*) For matched studies, give matching criteria and number of exposed and unexposed | N/A | |  |
| Variables | | 7 | | Clearly define all outcomes, exposures, predictors, potential confounders, and effect modifiers. Give diagnostic criteria, if applicable | Methods (sections on Collection and storage of placenta samples, Assay of placental inflammatory cytokine mRNA expression, Assessment of children’s cognitive development, Covariates); Additional file 1: Figure S1); Additional file 1: Table S3. | |  |
| Data sources/ measurement | | 8* | | For each variable of interest, give sources of data and details of methods of assessment (measurement). Describe comparability of assessment methods if there is more than one group | Methods (sections on Collection and storage of placenta samples, Assay of placental inflammatory cytokine mRNA expression, Assessment of children’s cognitive development, Covariates); Additional file 1: Figure S1); Additional file 1: Table S3. | |  |
| Bias | | 9 | | Describe any efforts to address potential sources of bias | Methods (“Assay of placental inflammatory cytokine mRNA expression”, “Assessment of children’s cognitive development”,“Statistical analysis” section. | |  |
| Study size | | 10 | | Explain how the study size was arrived at | Methods (“Study participants”) and Figure 1) | |  |
| Quantitative variables | | 11 | | Explain how quantitative variables were handled in the analyses. If applicable, describe which groupings were chosen and why | Methods (“Covariates”) and Additional file 1: Table S3) | |  |
| Statistical methods | | 12 | | (*a*) Describe all statistical methods, including those used to control for confounding | Methods (sections on “Covariates” and “Statistical analysis”) | |  |
|  |  |  |  | (*b*) Describe any methods used to examine subgroups and interactions | Methods (Statistical analysis, fifth paragraph) | |  |
|  |  |  |  | (*c*) Explain how missing data were addressed | Methods (Statistical analysis, second paragraph) | |  |
|  |  |  |  | (*d*) If applicable, explain how loss to follow-up was addressed | Methods (Statistical analysis, second paragraph) | |  |
|  |  |  |  | (*e*) Describe any sensitivity analyses | Methods (Statistical analysis, Seventh paragraph) | |  |
| Results | | | | |  | |  |
| Participants | | 13* | | (a) Report numbers of individuals at each stage of study—eg numbers potentially eligible, examined for eligibility, confirmed eligible, included in the study, completing follow-up, and analysed | Figure 1 | |  |
|  |  |  |  | (b) Give reasons for non-participation at each stage | Figure 1 and Methods (Study participants, Third paragraph) | |  |
|  |  |  |  | (c) Consider use of a flow diagram | Figure 1 | |  |
| Descriptive data | | 14* | | (a) Give characteristics of study participants (eg demographic, clinical, social) and information on exposures and potential confounders | Results (sections on “Basic characteristics of included participants” ) and Table 1 | |  |
|  |  |  |  | (b) Indicate number of participants with missing data for each variable of interest | Figure 1 and Additional file 1: Table S4 | |  |
|  |  |  |  | (c) Summarise follow-up time (eg, average and total amount) | Results (sections on “Distribution of placental cytokine expression and children’s cognitive performance” ) | |  |
| Outcome data | | 15* | | Report numbers of outcome events or summary measures over time | Results (sections on “Associations between placental inflammatory cytokines expression and children’s cognitive performance ” ) | |  |
| Main results | | 16 | | (*a*) Give unadjusted estimates and, if applicable, confounder-adjusted estimates and their precision (eg, 95% confidence interval). Make clear which confounders were adjusted for and why they were included | Results (sections on “Associations between placental inflammatory cytokines expression and children’s cognitive performance ”, “Sex-stratified associations between placental inflammatory cytokines expression and children’s cognitive performance”, “Associations between summary index of cytokines and children’s cognitive performance” ) , Table 2, Table 3, and Additional file 1 | |  |
|  |  |  |  | (*b*) Report category boundaries when continuous variables were categorized | N/A | |  |
|  |  |  |  | (*c*) If relevant, consider translating estimates of relative risk into absolute risk for a meaningful time period | N/A | |  |
| Other analyses | | 17 | | Report other analyses done—eg analyses of subgroups and interactions, and sensitivity analyses | Results (sections on “Sensitivity analyses”) | |  |
| Discussion | | | | | | |  |
| Key results | 18 | | Summarise key results with reference to study objectives | | Discussion (paragraph 1) | |  |
| Limitations | 19 | | Discuss limitations of the study, taking into account sources of potential bias or imprecision. Discuss both direction and magnitude of any potential bias | | Discussion (last paragraph) | |  |
| Interpretation | | 20 | | Give a cautious overall interpretation of results considering objectives, limitations, multiplicity of analyses, results from similar studies, and other relevant evidence | | Discussion (paragraph 2-paragraph 9) | |
| Generalisability | | 21 | | Discuss the generalisability (external validity) of the study results | Discussion (last paragraph) | | |
| Other information | | | | | | |  |
| Funding | | 22 | | Give the source of funding and the role of the funders for the present study and, if applicable, for the original study on which the present article is based | Declarations section | | |

*Give information separately for exposed and unexposed groups.

**Note:** An Explanation and Elaboration article discusses each checklist item and gives methodological background and published examples of transparent reporting. The STROBE checklist is best used in conjunction with this article (freely available on the Web sites of PLoS Medicine at http://www.plosmedicine.org/, Annals of Internal Medicine at http://www.annals.org/, and Epidemiology at http://www.epidem.com/). Information on the STROBE Initiative is available at http://www.strobe-statement.org.
